# Bayesian inference on stochastic gene transcription from flow cytometry data

**DOI:** 10.1093/bioinformatics/bty568

**Published:** 2018-09-08

**Authors:** Simone Tiberi, Mark Walsh, Massimo Cavallaro, Daniel Hebenstreit, Bärbel Finkenstädt

**Affiliations:** 1 Institute of Molecular Life Sciences, University of Zürich, Zürich, Switzerland; 2 Swiss Institue of Bioinformatics, University of Zürich, Zürich, Switzerland; 3 Department of Statistics, University of Warwick, Coventry, UK; 4 School of Life Sciences, University of Warwick, Coventry, UK

## Abstract

**Motivation:**

Transcription in single cells is an inherently stochastic process as mRNA levels vary greatly between cells, even for genetically identical cells under the same experimental and environmental conditions. We present a stochastic two-state switch model for the population of mRNA molecules in single cells where genes stochastically alternate between a more active ON state and a less active OFF state. We prove that the stationary solution of such a model can be written as a mixture of a Poisson and a Poisson-beta probability distribution. This finding facilitates inference for single cell expression data, observed at a single time point, from flow cytometry experiments such as FACS or fluorescence *in situ* hybridization (FISH) as it allows one to sample directly from the equilibrium distribution of the mRNA population. We hence propose a Bayesian inferential methodology using a pseudo-marginal approach and a recent approximation to integrate over unobserved states associated with measurement error.

**Results:**

We provide a general inferential framework which can be widely used to study transcription in single cells from the kind of data arising in flow cytometry experiments. The approach allows us to separate between the intrinsic stochasticity of the molecular dynamics and the measurement noise. The methodology is tested in simulation studies and results are obtained for experimental multiple single cell expression data from FISH flow cytometry experiments.

**Availability and implementation:**

All analyses were implemented in R. Source code and the experimental data are available at https://github.com/SimoneTiberi/Bayesian-inference-on-stochastic-gene-transcription-from-flow-cytometry-data.

**Supplementary information:**

Supplementary data are available at *Bioinformatics* online.

## 1 Introduction

This study aims at proposing a methodology for investigating transcription, i.e. the process by which mRNA transcripts are synthesized from genes in single cells. This process is fundamentally stochastic (Hebenstreit, 2013; Kim and Marioni, 2013; Raj *et al.*, 2006; Singh *et al.*, 2013) as it involves reactants present in low copy numbers and depends upon a series of events, whose timing is subject to natural variability (Delbrück, 1940; Kaern *et al.*, 2005; Kim and Marioni, 2013; Shahrezaei and Swain, 2008). Investigating stochasticity, or biological noise, in transcription is of particular interest as it could lead to an improved understanding of this essential cellular mechanism. Here, we develop a basic stochastic dynamic model regarding transcription and degradation events for the mRNA population of some gene of interest in single cells and show that the stationary distribution of the stochastic process can be written in a latent variable formulation which facilitates inference. In particular, we propose a two-state stochastic switch model where the gene alternates between a more and a less active state that in the sequel we refer to, for simplicity, as ON and OFF state, but we note that mRNA may be transcribed—albeit at a lower level—during the OFF state. In spite of its relative simplicity, this model can account for transcriptional bursts, corresponding to relatively short periods of time where high quantities of mRNA are transcribed. This phenomenon has been found to be typical of many genes and species (Dar *et al*., 2012; Golding *et al.*, 2005; Harper *et al.*, 2011; Raj *et al.*, 2006; Rajala *et al.*, 2010; So *et al.*, 2011; Suter *et al.*, 2011; Zopf *et al.*, 2013) although its underlying mechanism is still largely unclear and subject to ongoing research.

The fact that experimental measurement of gene expression is subject to measurement error gives rise to the concept that the mRNA population levels are unobserved, or *latent*. We make use of a pseudo-marginal approach (Andrieu and Roberts, 2009; Beaumont, 2003) to estimate the marginal likelihood of the noisy observations, integrating out the latent states. In order to infer the parameters of such a model, we develop a methodology for Bayesian posterior inference via Markov chain Monte Carlo (MCMC). Under the assumption of stationarity of the equilibrium solution, our method allows us to fit the proposed stochastic switch model to expression data obtained for a population of cells at a single time point, i.e. the kind of data typically available from fluorescence in situ hybridization (FISH) or FACS experiments. Inference is successfully tested in simulations studies. We provide an experimental data application of the inferential methodology where we analyse gene expression data in single cells, obtained via FISH flow cytometry experiments, for the human immunodeficiency virus type 1 (HIV-1) *env* gene, whose transcription is believed to occur in bursts. We infer the model parameters and compare two experimental conditions, where cells are stimulated at different levels, to gain insight into the transcriptional process and how it is affected by stimulation.

## 2 Two-state switch gene model

### 2.1 Model description

A basic model for gene expression assumes that, in each cell, transcription and degradation of mRNA molecules occur as a birth and death process with exponential waiting times, with constant rates *α* and *β*, respectively. It has been shown that the corresponding population of mRNA molecules in a cell at equilibrium follows a Poisson distribution (Paulsson, 2005; Raj *et al.*, 2006; Singh *et al.*, 2013). However, this model typically under-estimates the variability of the real biological mechanism and fails to explain the broadness of the distribution of the mRNA data, particularly for regulated genes. Indeed, the distribution of gene expression is often found to be overdispersed relative to the Poisson distribution, i.e. the variance is significantly larger than the mean (Munsky *et al*., 2012). A more realistic approach is hence provided by a two-state switch model which assumes that the gene stochastically alternates between ON and OFF states, with exponentially distributed waiting times with rates *k*_1_, for the change from OFF to ON, and *k*_0_, for the change from ON to OFF (Hebenstreit, 2013; Kim and Marioni, 2013; Munsky *et al.*, 2012; Peccoud and Ycart, 1995; Suter *et al.*, 2011; Wills *et al.*, 2013). In this model, it is assumed that the gene only transcribes mRNA in the ON state, while in the OFF state transcription is turned fully off. The resulting stationary distribution for the mRNA population has been derived to be the Poisson-beta distribution (Dattani and Barahona, 2017; Johnson *et al.*, 2005; Kim and Marioni, 2013), which can account for overdispersion as well as the occurrence of transcriptional bursts and thus highly improves upon modelling realism.

**Fig. 1.**
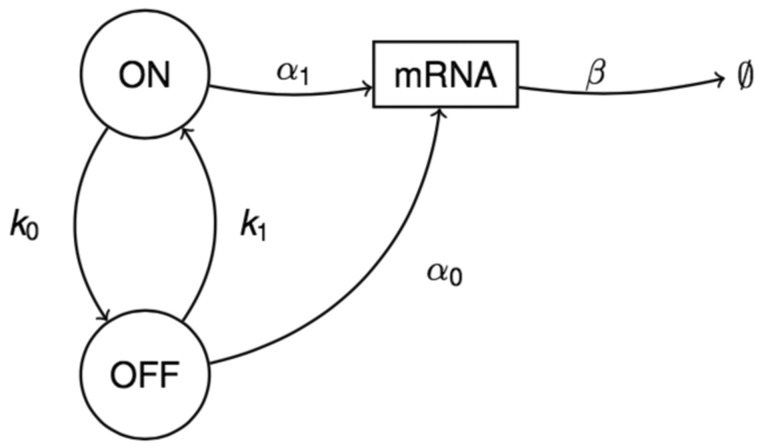
System for the proposed two-state switch model. The circles indicate the states of the gene, called ON or OFF, whilst the rectangle refers to the mRNA population. The parameters *k*_0_ and *k*_1_ represent the exponential rates at which the gene switches between the two states, while *α*_0_ and *α*_1_ are the transcription rates in the OFF and ON states, respectively, and *β* denotes the degradation rate

The assumption of zero transcription in the OFF state may be too restrictive in many cases (Hebenstreit *et al.*, 2011; Hey *et al.*, 2015). We therefore consider a two-state ON/OFF switch model where the gene may be transcribed into mRNA at two distinct rates, *α*_1_ and *α*_0_, such that $\alpha_{1}\geq \alpha_{0}\geq 0$, i.e. transcription in the OFF state is lower than in the ON state, but may occur at a positive rate (Kepler and Elston, 2001; Singh *et al.*, 2013; Thomas *et al.*, 2014). Figure 1 graphically illustrates this process. Degradation is assumed to happen at constant per molecule rate *β* and the states are subject to exponentially distributed waiting times, at rates *k*_1_ and *k*_0_, as above. The two-state switch model with zero transcription in the OFF state then corresponds to the sub-case where $\alpha_{0}=0$, while the simple one-state model can be obtained by setting $\alpha_{1}=\alpha_{0}$ or, equivalently, by assuming that the gene is transcribed at constant rate in one of the two states and setting $k_{0}=0$ or $k_{1}=0$.

Define $X={\left(X_{t}\right)}_{t\geq 0}$ as the $Z_{+}$-valued process representing the population of mRNA molecules in a cell, and $S={\left(S_{t}\right)}_{t\geq 0}$ as the {0, 1}-valued process indicating whether the gene is in ON state, if 1, or in OFF state, when 0. The model in Figure 1 can be represented by the Markov process $Z={\left(Z_{t}\right)}_{t\geq 0}=(S,X)$ with transition probabilities described by

(1) $$\begin{array}{c} P\left(Z_{t+dt}=\left(s\prime ,x\prime \right)\right)=\\ =\{\begin{array}{ll} \beta xdt+o(dt) & \;\text{if}\;\left(s\prime ,x\prime \right)=(s,x-1),\\ \left[\alpha_{1}s+\alpha_{0}(1-s)\right]dt+o(dt) & \;\text{if}\;\left(s\prime ,x\prime \right)=(s,x+1),\\ \left[k_{1}(1-s)+k_{0}s\right]dt+o(dt) & \;\text{if}\;\left(s\prime ,x\prime \right)=(1-s,x),\\ 0 & \text{otherwise}, \end{array} \end{array}$$

with $Z_{t}=(s,x)$ indicating the state of the system at time *t*.

Next, using the result by Singh *et al.* (2013) we shall prove that the distribution of the mRNA population at equilibrium can be equivalently represented by a mixture of a Poisson and a Poisson-beta distribution. This result facilitates the construction a Bayesian inference algorithm to sample directly from the equilibrium distribution of the mRNA population.

### 2.2 Stationary distribution

Singh *et al.* (2013) show that the mRNA counts from the two-state model in (1) have the following stationary distribution
(2)
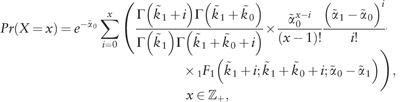

where 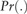 indicates the probability operator, *X* denotes the random variable (rv) representing the mRNA counts, Γ refers to the gamma function and _1_*F*_1_ is the confluent hypergeometric function of the first kind. We note that the degradation parameter *β* is not identifiable as it appears only in combination with other parameters. In the sequel we consider a reparameterization where the remaining kinetic parameters are scaled with respect to the degradation rate, i.e. ${\tilde{\alpha }}_{0}=\frac{\alpha_{0}}{\beta }$, ${\tilde{\alpha }}_{1}=\frac{\alpha_{1}}{\beta }$, ${\tilde{k}}_{1}=\frac{k_{1}}{\beta }$ and ${\tilde{k}}_{0}=\frac{k_{0}}{\beta }$. Inference focuses on the scaled parameters ${\tilde{\alpha }}_{0},\;{\tilde{\alpha }}_{1},\;{\tilde{k}}_{1}$ and ${\tilde{k}}_{0}$. The following theorem states that the stationary probability distribution for *X* can be written as a mixture of a Poisson and a Poisson-beta density, which can usefully be exploited for inference.

Theorem: The density in (2) can be associated with the following latent variable structure,

(3) X=A+B

where

(4) $$A∼\text{Pois}\left(({\tilde{\alpha }}_{1}-{\tilde{\alpha }}_{0})P\right)$$

(5) $$P∼\text{Beta}\left({\tilde{k}}_{1},{\tilde{k}}_{0}\right)$$

(6) $$B∼\text{Pois}({\tilde{\alpha }}_{0}),$$

where *Pois*(*x*) indicates the Poisson rv with mean *x* and variance *x* and *Beta*(*a*, *b*) represents the beta rv with mean a/(a+b) and variance $\frac{ab}{{\left(a+b\right)}^{2}(a+b+1)}$. The probability functions for *A* (Johnson *et al*., 2005) and *B* are
(7)
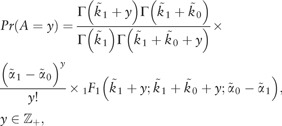

and

(8) $$Pr(B=z)=e^{-{\tilde{\alpha }}_{0}}\frac{{\tilde{\alpha }}_{0}^{z}}{z!},z\in Z_{+}.$$

Proof: Since *X* is defined as the summation of *A* and *B*, *X *=* x* is obtained when (A=a,B=b) with (a,b)∈{(0,x),(1,x−1),…,(x−1,1),(x,0)}. Furthermore, given *A* and *B* are independent Pr(A=a,B=b)=Pr(A=a)Pr(B=b) conditionally on parameters. Hence, the probability density for *X* can be obtained, via the discrete convolution formula, as

(9) $$Pr(X=x)=\sum_{i=0}^{x}Pr(A=i)Pr(B=x-i),\;x\in Z_{+}$$

(10)
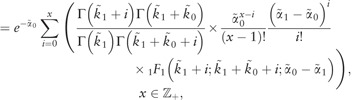

which corresponds to the formula in (2). The formulation in (10) follows from (9) by replacing the probabilities of *A* and *B* with their formulae (7) and (8), respectively. This completes the proof of the theorem.

Hence, we have shown that *X* can be written as the summation of *A* and *B*, as in (3)–(6). Furthermore, since the summation of two independent Poisson rvs is again Poisson, we can rewrite the distribution of *X* as

(11) $$X|P∼\text{Pois}\left(\left({\tilde{\alpha }}_{1}-{\tilde{\alpha }}_{0}\right)P+{\tilde{\alpha }}_{0}\right),\;\text{with}\;P∼\text{Beta}\left({\tilde{k}}_{1},{\tilde{k}}_{0}\right).$$

We note that *P* can be interpreted as the probability that the gene is in the ON state (Johnson *et al*., 2005), and its mean represents the average time the gene spends in the ON state. Explicit expressions for the mean and variance of *P* and *X* are derived in the Supplementary Section S2.3. To compute *Pr*(*X*) in (2), _1_*F*_1_ needs to be estimated numerically, which is challenging (Kim and Marioni, 2013; Muller, 2001). However, the decomposition of *X* in (11) provided by the theorem shows that this computation can be avoided by taking advantage of the latent variable structure to sample *X* without the need to explicitly compute *Pr*(*X*).

## 3 Inference

### 3.1 Measurement equation

As the mRNA molecule count cannot be observed exactly, we assume that the observation for cell *i*, *Y_i_*, is proportional to the actual population of mRNA, *X_i_*, and that the measurement process is perturbed by measurement noise. In our FISH flow cytometry experimental data, observations coming from a sample of *N* cells, $Y=(Y_{1},\ldots ,Y_{N})$, are assumed to be linked to the original mRNA levels, denoted by $X=(X_{1},\ldots ,X_{N})$, via a measurement equation which involves a proportionality constant, *κ*, and additive Gaussian measurement error, which we assume to be independently and identically distributed (iid):

(12) $$Y_{i}=\kappa X_{i}+\epsilon_{i},\;\text{for}\;i=1,\ldots ,N,$$

with $\epsilon_{i}∼N\left(\mu_{\epsilon },\sigma_{\epsilon }^{2}\right)$, where $\epsilon_{i}$ represents the measurement error for the *i*th cell and N(a,b) is the normal rv with mean *a* and variance *b*. In the analysis of the background noise data, described in Supplementary Section S2.2, we show that the normal distribution approximates the background error of our experimental data reasonably well. We assume that $\mu_{\epsilon }$ is positive. The reason for this is that, although ideally the fluorescence probes should bind specifically to the mRNA of interest only, some probes will bind in an unspecific way and the cells can exhibit autofluorescence. Furthermore, since the measurement process strongly amplifies the fluorescence signal from the original populations of mRNA molecules, we also assume κ>1.

We note that, due to the measurement process, the unobservable mRNA population in each cell, *X_i_*, is a latent state variable. The marginal likelihood of the observation for the *i*th cell, given the parameter vector $\theta ={\left({\tilde{\alpha }}_{0},{\tilde{\alpha }}_{1},{\tilde{k}}_{1},{\tilde{k}}_{0},\kappa ,\mu_{\epsilon },\sigma_{\epsilon }\right)}^{T}$, is obtained by integrating over the latent states as

(13) $$\begin{array}{l} Pr(Y_{i}=y_{i}|\theta )=\int_{Z_{+}}Pr(Y_{i}=y_{i}|X_{i}=x,\theta )Pr(X_{i}=x|\theta )dx,\\ =\sum_{x=0}^{\infty }Pr(Y_{i}=y_{i}|X_{i}=x,\theta )Pr(X_{i}=x|\theta ). \end{array}$$

In practice, we approximate (13) by drawing a finite sample of size *S* from (11), $z_{i}=\left(z_{i}^{\left(1\right)},\ldots ,z_{i}^{\left(S\right)}\right)$, to compute the following unbiased approximation:

(14) $$\hat{f}(y_{i}|\theta )=\sum_{s=1}^{S}\frac{P\left(Y_{i}=y_{i}|X_{i}=z_{i}^{\left(s\right)},\theta \right)}{S}.$$

In order to approximate the densities of all observations, we should draw *N* samples of size *S*, $z_{1},\ldots ,z_{N}$, which would be computationally prohibitive. At the same time, in spite of the independent and identically distributed (iid) nature of the data, using the same sample $z=\left(z^{\left(1\right)},\ldots ,z^{\left(S\right)}\right)$ for all *N* data points would lead to a biased estimator. Here, we use a recently developed estimator which allows us to employ the same *S* particles for all observations while preserving unbiasedness. The method is illustrated in detail in the Supplementary Section S1.1. We combine this calculation with a pseudo-marginal method where, in the MCMC algorithm, these unbiased estimates replace the original marginal probabilities.

We note that our approach explicitly allows for two sources of noise, namely the intrinsic stochasticity due to the biological noise, inherent in the molecular processes associated with transcription and degradation, and the measurement noise, which is not part of the molecular dynamics. The approach outlined below does not rely on the Gaussianity assumption of the measurement noise, and can be extended in a straightforward way to other distributional specifications for the measurement error. An alternative to the pseudo-marginal approach is to explicitly perform a data augmentation procedure to sample the latent states together with the other parameters of the model. We implemented and tested both methods on simulated data with the conclusion that the former resulted in improved mixing and convergence of the posterior chains while the data augmentation procedure with two layers per cell, i.e. *P_i_* and *X_i_*, led to a highly correlated multidimensional posterior space, which was much more challenging to explore.

### 3.2 A hierarchical model for biological replicates

As will be shown in Section 4.1, the experimental data available was collected in four biological replicates, each containing a multitude of single cell observations. The full data of an experiment with *K* replicates is $Y=\left(Y^{\left(1\right)},\ldots ,Y^{\left(K\right)}\right)$ with $Y^{\left(k\right)}={\left(Y_{1}^{\left(k\right)},\ldots ,Y_{N_{k}}^{\left(k\right)}\right)}^{T}$ representing the *N_k_* observations available for the *k*th replicate, k=1,…,K. In our case *K* = 4. The hierarchical measurement equation, relating the observations to the latent mRNA populations is

(15) $$Y_{i}^{\left(k\right)}=\kappa^{\left(k\right)}X_{i}^{\left(k\right)}+\epsilon_{i}^{\left(k\right)},\;\text{for}\;i=1,\ldots ,N_{k}\;\text{and}\;k=1,\ldots ,K,$$

with $\epsilon_{i}^{\left(k\right)}∼N\left(\mu_{\epsilon }^{\left(k\right)},\sigma_{\epsilon }^{2\;(k)}\right)$.

Define the hierarchical parameter vector for the *k*th replicate as

$$\theta^{\left(k\right)}={\left({\tilde{\alpha }}_{0}^{\left(k\right)},{\tilde{\alpha }}_{1}^{\left(k\right)},{\tilde{k}}_{1}^{\left(k\right)},{\tilde{k}}_{0}^{\left(k\right)},\kappa^{\left(k\right)},\mu_{\epsilon }^{\left(k\right)},\sigma_{\epsilon }^{\left(k\right)}\right)}^{T}.$$

**Fig. 2.**
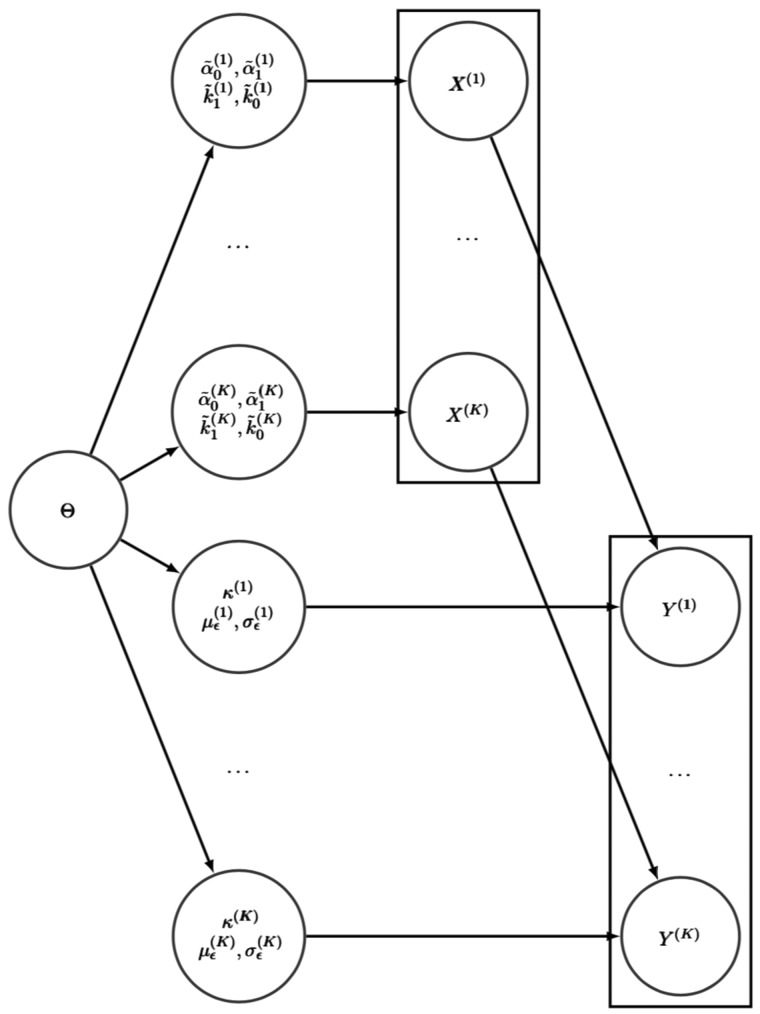
Graphical model for the hierarchical system. On the left side the hyperparameters Θ generate the hierarchical parameters. Given the kinetic hierarchical parameters, the latent states $X^{\left(1\right)},\ldots ,X^{\left(K\right)}$ are drawn from (2). These, together with the hierarchical measurement equation parameters, generate the observed data $Y^{\left(1\right)},\ldots ,Y^{\left(K\right)}$

Bayesian hierarchical modeling (Finkenstädt *et al*., 2013; Gamerman and Lopes, 2006; Hey *et al.*, 2015) provides a natural framework for pooling data from several experiments whilst quantifying variation between biological replicates in a statistically rigorous way. In contrast to assuming that a replicate *k* is described by exactly the same value of the parameter vector, in a hierarchical model it is a random sample from a joint distribution $p\left(\theta^{\left(k\right)}|\Theta \right)$ with $\Theta =(\Theta_{1},\ldots ,\Theta_{q})$, where *q* is the number of parameters in $\theta^{\left(k\right)}$, *q* = 7 in our case, and each $\Theta_{j}={\left(\mu_{j},\tau_{j}\right)}^{T},\;j=1,\ldots ,q$, is a hyperparameter vector quantifying the mean and precision of the *j*th parameter across the replicates. The graphical model for the hierarchical system used is shown in Figure 2.

Assuming that replicates are independent, the full likelihood for all cells in the experiment is

(16) $$L(\theta ;Y)=\prod_{k=1}^{K}L\left(\theta^{\left(k\right)};y^{\left(k\right)}\right)=\prod_{k=1}^{K}\prod_{i=1}^{N_{k}}P\left(Y_{i}^{\left(k\right)}|\theta^{\left(k\right)}\right)$$

with $\theta =\left(\theta^{\left(1\right)},\ldots ,\theta^{\left(K\right)}\right)$ denoting the matrix of hierarchical parameter vectors and where $y^{\left(k\right)}={\left(y_{1}^{\left(k\right)},\ldots ,y_{N_{k}}^{\left(k\right)}\right)}^{T}$ indicates the realization of $Y^{\left(k\right)}$. In the MCMC algorithm, we replace the intractable likelihood $L\left(\theta^{\left(k\right)};y^{\left(k\right)}\right)$, which involves a latent state for the unknown mRNA population of every cell, with an unbiased estimate as in (14).

Let $p(\theta |\Theta )=\prod_{k=1}^{K}\prod_{j=1}^{q}p\left(\theta_{j}^{\left(k\right)}|\Theta_{j}\right)$ denote the prior distribution of θ and $p(\Theta )=\prod_{j=1}^{q}p(\Theta_{j})$ the prior distribution for the hyperparameter Θ. The posterior distribution of the parameters given the data is then proportional to

(17) p(θ,Θ|Y)∝L(θ;Y)p(θ|Θ)p(Θ).

In the hierarchical model we wish to infer upon the posterior p(θ,Θ|Y) which is achieved by formulating an appropriate MCMC algorithm that samples from it.

### 3.3 Prior distributions

For all our hierarchical parameters we use a log-normal prior distribution

(18) $$p\left(\theta_{j}^{\left(k\right)}|\Theta_{j}\right)∼\;\text{log}\;N\left(\mu_{j},\frac{1}{\tau_{j}}\right),$$

where log N(a,b) denotes the log-normal distribution with mean *a* and variance *b*, which has $R^{+}$ as support. As $\kappa^{\left(k\right)}>1$ we assume a truncated log-normal prior distribution with support in (1,∞). Regarding the hyperparameters we assume the well known normal-gamma conjugate prior model (Gamerman and Lopes, 2006)

(19) $$\mu_{j}|\tau_{j}∼N\left(a_{j},\frac{b_{j}}{\tau_{j}}\right)\;\text{and}\;\tau_{j}∼G(c_{j},d_{j}),\;\text{for}\;j=1,\ldots ,q,$$

where *a_j_*, *b_j_*, *c_j_* and *d_j_* are the hyperprior parameters and G(a,b) is the gamma distribution with mean $\frac{a}{b}$ and variance $\frac{a}{b^{2}}$. The choice of the prior and hyperprior distributions leads to conjugate forms of the conditional posterior distributions for the hyperprior parameters, which allow us to sample using a corresponding Gibbs sampler (Hey *et al*., 2015). The hyperparameters *μ*_5_ and *τ*_5_ are sampled via a Metropolis–Hasting sampler, due to the truncation of the log-normal distribution for *κ*. We set *a_j_* = 0, $b_{j}=10^{4},\;c_{j}=0.001$ and $d_{j}=0.001$, which correspond to a vague normal prior with zero mean for the hypermean, *μ_j_*, and a vague gamma prior for the hyperprecision, *τ_j_*, with mean 1 and variance 10^3^. The hyperparameters for the measurement error, *μ*_6_, *μ*_7_, *τ*_6_ and *τ*_7_, are not sampled: the hierarchical parameters $\mu_{\epsilon }^{\left(k\right)}$ and $\sigma_{\epsilon }^{\left(k\right)}$ are assumed to follow a constant informative prior, which is distinct for each replicate, k=1,…,K, and matches the results we obtained from an additional analysis of background noise data shown in the Supplementary Section S2.2.

### 3.4 Markov chain Monte Carlo

We develop a Metropolis–within-Gibbs algorithm (Hastings, 1970; Metropolis and Ulam, 1949; Metropolis *et al.*, 1953) where parameters are alternately sampled from their conditional distributions: the hyperparameters Θ|θ are sampled from a Gibbs sampler; the hierarchical parameters θ|Θ,Y are sampled, separately for each replicate, via a Metropolis algorithm in two blocks, one for $\left({\tilde{\alpha }}_{0},{\tilde{\alpha }}_{1},{\tilde{k}}_{1},{\tilde{k}}_{0},\kappa \right)$ and one for $\left(\mu_{\epsilon },\sigma_{\epsilon }\right)$. This particular choice was motivated by maximizing the correlation of the parameters within each block such that correlated parameters are updated jointly. Such strategy was found to significantly improve mixing of the posterior chains. The proposal values for the Metropolis algorithm are sampled via an adaptive random walk (ARW) scheme (Haario *et al*., 2001) where during any Metropolis step, the actual likelihood, which involves a latent state for the mRNA population, is replaced by an unbiased estimate as in (14). The details of the sampling scheme are described in the Supplementary Section S1.2.

### 3.5 Simulation study

In order to assess the performance of our inferential methodology we carried out a simulation study where we simulated six datasets, each composed of 4 replicates of 1000 independent observations, the same size as the experimental data used. The parameter values were chosen approximately such that they give rise to densities that are broadly similar to the ones observed for the experimental data. The details of the simulation study are provided in the Supplementary Section S1.3. The parameter values used are reported in the Supplementary Tables S1 and S2 and the simulated densities are shown in Supplementary Figure S1. For each simulated dataset, we apply our Bayesian hierarchical estimation algorithm to sample from the posteriors of the model parameters as described above. In each simulation study, the MCMC algorithm was run for $6\times 10^{5}$ iterations where the first 10^5^ iterations were discarded as burn-in. We computed the highest posterior density (HPD) credible intervals (CIs) via the *HPDinterval* function of the R (R Core Team, 2016) package *coda* (Plummer *et al*., 2016). Table 1 displays the empirical coverages of the 0.90 and 0.95 level HPD CIs for the hierarchical and hyper parameters, respectively. On average 98.2 and 98.8% of the hierarchical parameters fall in the 0.90 and 0.95 level HPD CIs, respectively; while all hypermean and hyperprecision parameters fall in the respective 0.90 and 0.95 level HPD CI. We hence conclude that the algorithm performs well in retrieving the unknown parameters.

**Table 1.** Coverage of the 0.90 and 0.95 level HPD CIs for the hierarchical parameters, out of 24 (6 simulations of 4 replicates each)

Level	${\tilde{\alpha }}_{0}$	${\tilde{\alpha }}_{1}$	${\tilde{k}}_{1}$	${\tilde{k}}_{0}$	*κ*	$\mu_{\epsilon }$	$\sigma_{\epsilon }$	Average (%)
0.90	24	24	21	24	24	24	24	98.2
0.95	24	24	22	24	24	24	24	98.8

## 4 Experimental data analysis

### 4.1 Data description

As a case study of the proposed methodology we analyse single cell expression data obtained from a modified version of HEK293 cells containing a version of the HIV-1 *env* gene under the control of a *tetracycline* inducible promoter (Damgaard *et al*., 2008). The mRNA levels are observed, separately for each cell, via FISH flow cytometry where the native mRNA is tagged with fluorescent labelled oligos, which are short nucleotide sequences designed to bind specifically to the mRNA of interest. A laser is then used to induce these tagged mRNAs to emit light. The measurement procedure is illustrated in Supplementary Figure S2. The BD FACSDiva^TM^ software, of the BD LSRFortessa^TM^ cell analyzer, is used to measure the overall light intensity in each cell. While about 10 000 observations were detected in each replicate we find that the distribution of the data is already accurately approximated by using 1000 observations with very marginal loss of information and we therefore present results here using data from a randomly selected subset of 1000 observations. The HIV-1 *env* gene under study is observed at two levels, 5 and 10ng/ml, of induction by *tetracycline*. In each of the two experimental conditions, data are collected in the same four biological replicates. Supplementary Figure S6 shows the densities obtained from the experimental data in each replicate. Interest lies in inferring the kinetic parameters of the model and in studying the effect of *tetracycline* on the system.

### 4.2 Inference

We apply the hierarchical Bayesian methodology described in Section 3 separately to each dataset corresponding to the two experimental conditions. The MCMC algorithm was run for at least $6\times 10^{5}$ iterations, the first 10^5^ of which were discarded as burn-in. We use the Heidelberger and Welch convergence diagnostic (Heidelberger and Welch, 1981, 1983), via the *heidel.diag* function of the R package *coda* (Plummer *et al*., 2016), to test for the stationarity of each chain and automatically assess its burn-in period. We apply the convergence test to all hierarchical and hyperparameters and found that none of them were rejected at the 1% significance level. For one parameter the estimated burn-in was larger i.e. $1.8\times 10^{5}$. In this case we ran the MCMC for longer in order to increase the burn-in to the one estimated by *heidel.diag* and keep the following $5\times 10^{5}$ iterations as our posterior sample. For the hyperparameters, we use a thinning factor of 100, while we keep all iterations from the hierarchical parameters. After having removed the burn-in period, we use the *ess* function of the *mcmcse* R package (Flegal *et al*., 2017) to compute the effective sample size (ess) of every posterior sample, i.e. the size of an iid sample with the same variance as the chain considered. All the ess estimates are above 170 for the hyperparameters, with an average ess of about 2500, and above 785 for the hierarchical parameters, with an average ess of approximately 10 000. To appreciate the convergence and mixing of the algorithm, Supplementary Figures S10 and S11 show the thinned chains of two re-parametrizations of the hierarchical parameters representing the mean and standard deviation of $y^{\left(1\right)},\ldots ,y^{\left(K\right)}$. The horizontal lines represent the sample mean and standard deviation observed in the respective experimental data and always fall in the central area of the posterior chains.

### 4.3 Results

**Fig. 3.**
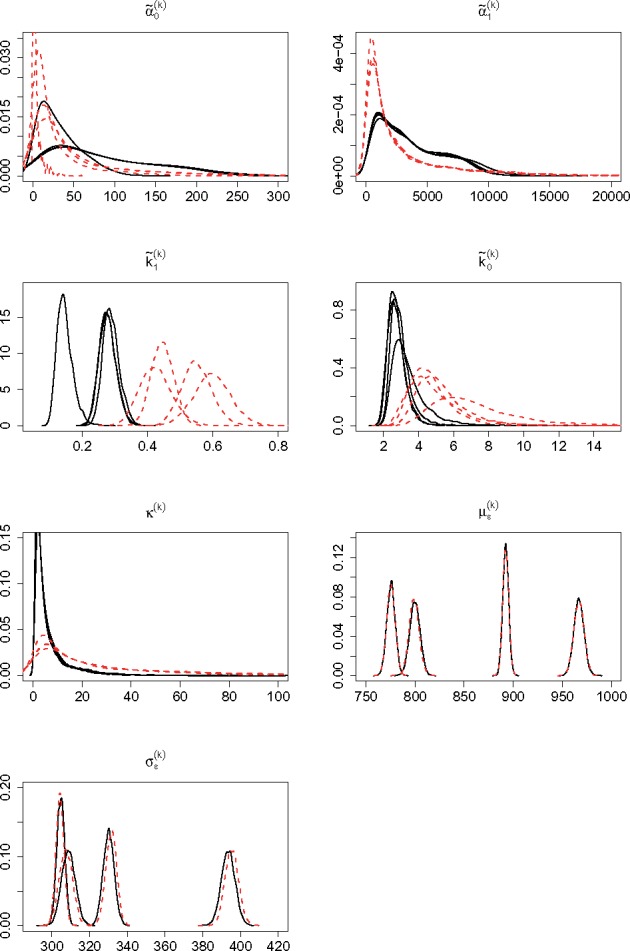
Posterior densities for the hierarchical parameters. The black solid and red dotted lines refer to cells stimulated with 5 and 10 ng/ml of *tetracycline*, respectively

**Fig. 4.**
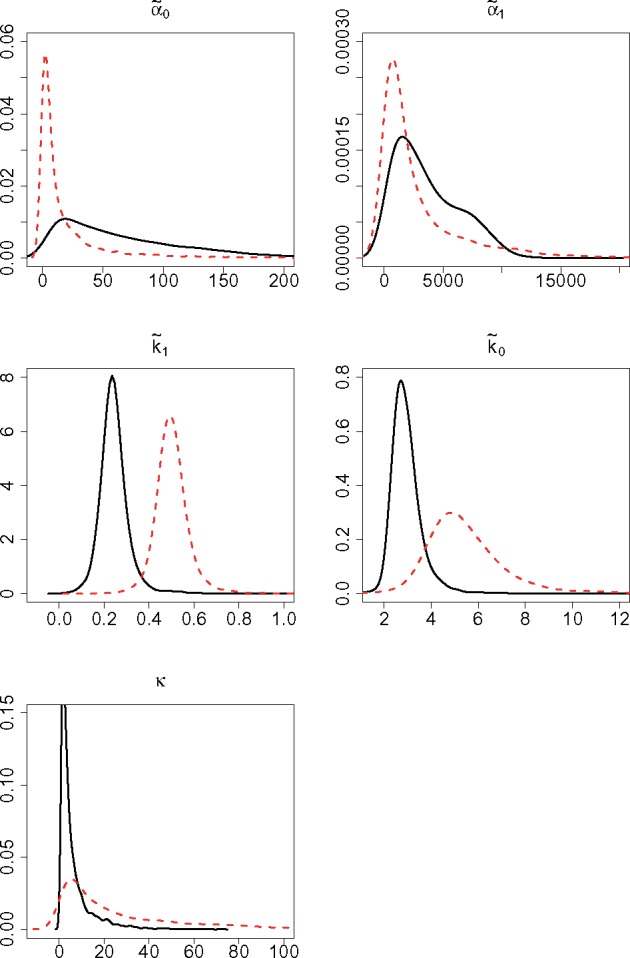
Posterior densities for the exponential of the hypermean parameters, $e^{\mu_{1}},\ldots ,e^{\mu_{5}}$, representing the posterior median of the respective hierarchical parameters, i.e. ${\tilde{\alpha }}_{0},{\tilde{\alpha }}_{1},{\tilde{k}}_{1},\;{\tilde{k}}_{0}$ and *κ*. The black solid and red dotted lines refer to cells stimulated with 5 and 10 ng/ml of *tetracycline*, respectively

Figure 3 shows the posterior densities for the hierarchical parameters in both experimental conditions, while Figure 4 shows the estimated posterior densities for the exponential transformations of the hypermean parameters which, in a log-normal distribution, represent the posterior modes of the respective hierarchical parameters. Further estimation details are provided in Supplementary Table S5, which gives the 0.95 level HPD CIs for the exponential of the hypermean parameters, in Supplementary Tables S6 and S7, that list the 0.95 level HPD CIs for the hierarchical parameters and some reparametrizations of these, and in Supplementary Figure S9, which shows the posterior densities for the hyperprecision parameters.

Inference reveals insights into the transcriptional process and how it is affected by an increased level of stimulation. We notice that the posterior densities for the measurement error parameters, as well as for the proportionality constant *κ*, are mostly unchanged between experimental conditions. This is expected as they are associated with the measurement process which in principle remained unchanged between experiments. Naturally, the measurement error parameters are very similar across conditions also due to the informative prior used. Regarding the kinetic parameters we note that, the transcription rates, ${\tilde{\alpha }}_{0}$ and ${\tilde{\alpha }}_{1}$, only show minor variations, while both switch rates, and particularly ${\tilde{k}}_{1}$, clearly increase with the higher level of stimulus. In particular, the fold change between conditions of the posterior modes of ${\tilde{k}}_{1}$ and ${\tilde{k}}_{0}$, i.e. $e^{\mu_{3}}$ and $e^{\mu_{4}}$, is 2.0 and 2.3, respectively. It hence appears that, in cells stimulated at a higher level of *tetracycline*, the speed of both ON and OFF switching increases.

We also compute the coefficient of variation (CV), i.e. the ratio of the standard deviation to the mean, of the hierarchical parameters across replicates, to study how parameters vary between biological replicates. Table 2 reports the posterior means of the CVs: in both conditions, the measurement error parameters and ${\tilde{\alpha }}_{1}$ show the smallest variation, the switch rates exhibit more variability, while ${\tilde{\alpha }}_{0}$ clearly is the most variable parameter between replicates. The same indication is also evident when looking at how the posterior densities of the hierarchical parameters vary between replicates in Figure 3.

**Table 2.** Posterior mean of the CV of the hierarchical parameters across replicates

***Tetracycline* (**ng/ml)	${\tilde{\alpha }}_{0}$	${\tilde{\alpha }}_{1}$	${\tilde{k}}_{1}$	${\tilde{k}}_{0}$	*κ*	$\mu_{\epsilon }$	$\sigma_{\epsilon }$
5	0.39	0.11	0.28	0.15	0.13	0.10	0.12
10	0.78	0.19	0.18	0.28	0.21	0.10	0.13

**Fig. 5.**
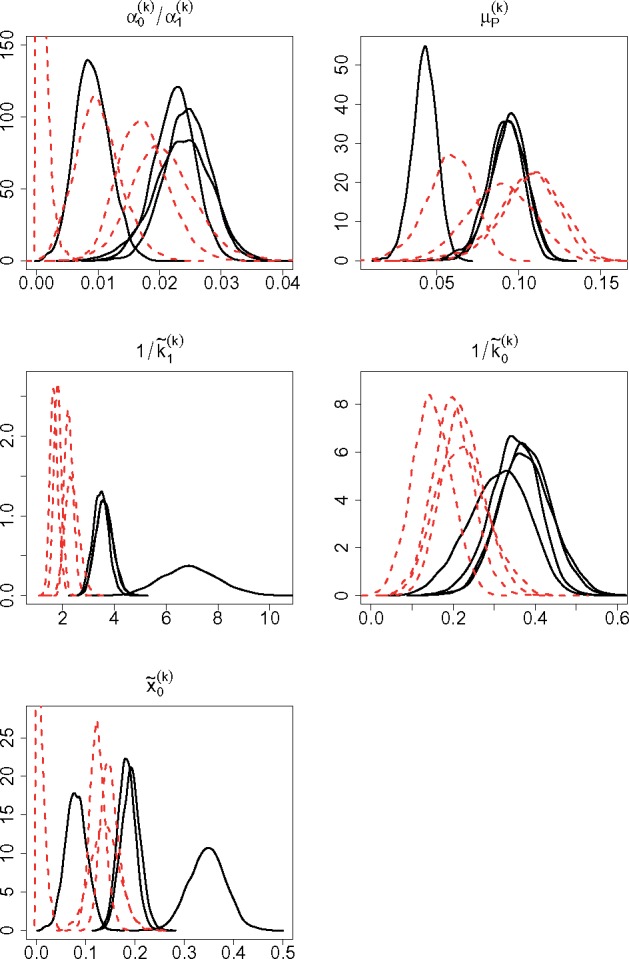
Posterior densities for the following reparametrizations of the hierarchical parameters: $\frac{\alpha_{0}^{\left(k\right)}}{\alpha_{1}^{\left(k\right)}},\;\mu_{P}^{\left(k\right)},\;1/{\tilde{k}}_{1}^{\left(k\right)},\;1/{\tilde{k}}_{0}^{\left(k\right)}$ and ${\tilde{x}}_{0}^{\left(k\right)}$, with k=1,…,4, where ${\tilde{x}}_{0}^{\left(k\right)}=\frac{\alpha_{0}^{\left(k\right)}\left(1-\mu_{P}^{\left(k\right)}\right)}{\alpha_{0}^{\left(k\right)}\left(1-\mu_{P}^{\left(k\right)}\right)+\alpha_{1}^{\left(k\right)}\mu_{P}^{\left(k\right)}}$ represents the fraction of mRNA which is transcribed from the OFF state. The black solid and red dotted lines refer to cells stimulated with 5 and 10 ng/ml of *tetracycline*, respectively

Figure 5 shows some reparametrizations of the hierarchical parameters which allow us to gain further insight into the transcriptional process. We note that the ratio between ${\tilde{\alpha }}_{0}$ and ${\tilde{\alpha }}_{1}$ falls between 0 and 0.04, which confirms that transcription in the OFF state usually is non-zero and that our assumption of positive transcription in both states is more realistic for this gene. Nonetheless, it also highlights the finding that transcription in the active state is orders of magnitude higher than in the inactive one. Studying the mean of *P*, *μ_P_*, allows us to compare the overall time the gene spends in the ON state between conditions. It appears that, for an increasing dose of *tetracycline*, although both switches are accelerated, there is some considerable variation between replicates and no discernible difference in the time the gene spends in the more active state. We also find that the gene spends between approximately 3 and 14% of the time in the ON state, while most of the time they are OFF. Despite ${\tilde{\alpha }}_{0}$ being much smaller than ${\tilde{\alpha }}_{1}$, since the gene is mostly OFF, we find that a significant fraction of mRNA is transcribed from the OFF state (Table 3 and Fig. 5, bottom panel). The asymmetry in timing along with the large difference in the associated transcription rates is responsible for the dynamic appearance of short and intense bursts, and the findings here are consistent with results obtained from fitting switch-type stochastic models to single cell reporter imaging time series data for other genes (Harper *et al*., 2011; Hey *et al.*, 2015). In particular, Dunham *et al.* (2017) further characterize this asymmetry by showing that switches from the OFF to the ON states are typically abrupt and result in short and intense bursts which are followed by a gradual deactivation of the gene. The estimation results for the inverted switch rates (Fig. 5, middle panels), which correspond to the average time the gene spends in each state, confirm that a higher level of stimulation leads to a faster switching behaviour. Note that the time unit here is the degradation rate. This appears to be a strong result as there are clear differences between the two levels of stimulus, particularly for the ON switching.

**Table 3.** Posterior mean for the fraction of mRNA which is transcribed from the OFF state in each replicate for both experimental conditions

***Tetracycline* (**ng/ml)	Replicate			
	1	2	3	4
5	0.19	0.08	0.35	0.18
10	0.15	0.01	0.14	0.12

For each replicate and experimental condition, we simulated the observed data, $Y^{\left(k\right)}$, for k=1,…,4, from 100 posterior values of the hierarchical parameters $\theta^{\left(k\right)}$: in all cases the simulated densities closely match the experimental data, showing that the parameter values inferred, and the model used, are able to reproduce very similar patterns as those experimentally observed (Supplementary Fig. S8).

The latent population of mRNA in single cells is estimated to occupy a range between a few tens to a few hundreds of molecules, while the ratio between variance and mean is inferred to be orders of magnitude bigger than 1 (the lower bounds of all 0.95 level HPD CIs are bigger than 10), which highlights the large degree of overdispersion observed for gene expression in single cells (details in Supplementary Fig. S7 and HPD CIs in Supplementary Tables S6 and S7).

## 5 Conclusions

We propose a stochastic gene expression model that allows for transcriptional switching between two states, where transcription in the so called OFF state is less active than in the ON state, but may occur at a positive rate. While approaches exist to fit this system, and indeed more complex types of switch models, to single cell time series imaging data on gene expression (Featherstone *et al*., 2016; Harper *et al.*, 2011; Hey *et al.*, 2015), the aim here is for such a model to be fitted to single cell expression data from flow cytometry experiments such as FACS or FISH, which only report gene expression at a single point in time. We show that the stationary distribution of the stochastic process can be decomposed as a mixture of a Poisson and a Poisson-beta distribution, a latent structure that greatly facilitates inference as it allows one to sample the population of mRNA molecules at equilibrium instead of having to approximate its density numerically. We also formulate a process exhibiting measurement error, which introduces a latent state for the mRNA population, and develop a pseudo-marginal likelihood approach to integrate over the latent states. In order to infer the model parameters, we develop a methodology for Bayesian posterior inference via MCMC, where we embed the model into a Bayesian hierarchical structure, which allows us to quantify the variability between biological replicates. The methodology is validated in simulation studies and applied to experimental single cell FISH flow cytometry expression data obtained from a version of the HIV-1 *env* gene under the control of a *tetracycline* inducible promoter. We find strong evidence that transcription mostly happens in short and intense bursts, where the gene spends most of the time in the less active state, and only switches for a short time into a more active state, the latter being characterized by a much larger transcription rate. For increasing level of stimulus, the transcription rates are mostly unchanged, while there is a significantly increased speed of switching in both states.

Further analyses are currently being performed to compare more experimental conditions and to investigate how transcription varies during the life cycle of a cell. We note that Harper *et al.* (2011) developed methods to reconstruct transcription dynamics from two loci in real time in single cells and were able to provide evidence for the existence of a refractory period in the inactivation phase of gene transcription. This finding has since been confirmed as an important ubiquitous property of genes (see, e.g. Molina *et al.,* 2013). Hence, a potential aspect to address in future work is to investigate the presence of such a period by introducing an intermediate state between the OFF and ON states, which would allow to model gene activation in two steps. Inferring this from flow cytometry experimental data alone might pose challenges to parameter identifiability, in particular if measurement error modelling is included. On the other hand, an approach combining time series reporter imaging with flow cytometry expression data may be a promising way forward to fit these kinds of models to experimental data in order to study in more detail the processes involved in transcription and transcriptional regulation.

## Supplementary Material

Supplementary Data
