# Supplementary material for: Bayesian inference on stochastic gene transcription from flow cytometry data
Source: Bioinformatics. 2018 Sep 8;34(17):i647–55. doi: 10.1093/bioinformatics/bty568 (PMC6129284; doi:10.1093/bioinformatics/bty568)
Supplement: Supplementary Data [file bty568_supplementary_material.pdf]

SUPPLEMENTARY MATERIAL

Bayesian inference on stochastic gene transcription from flow cytometry data

Simone Tiberi, Mark Walsh, Massimo Cavallaro, Daniel Hebenstreit and Bärbel Finkenstädt.

Contents

S1 Methods **1**
S1.1 Unbiased approximation of products of integrals 1
S1.2 MCMC algorithm 2
S1.3 Simulation study set-up 5

S2 Experimental data **8**
S2.1 Measurement process 8
S2.2 Exploratory analysis of the measurement error 8
S2.3 Mean and variance of P, X and Y 12
S2.4 Additional Figures and Tables 14

S1 Methods

S1.1 Unbiased approximation of products of integrals

We use a pseudo-marginal approach where the marginal probability of the i-th observation, $P(Y_i = y_i|\theta)$, is approximated by an unbiased estimate, $\widehat{f}(y_i|\theta)$.

Given the independent and identically distributed (iid) nature of the data, the likelihood for a sample of N observations $y = (y_1, \dots, y_N)$, is defined by:

$$L(\theta|y) = \prod_{i=1}^{N} P(Y_i = y_i|\theta). \quad \text{(S1)}$$

The standard way to approximate (S1) is to generate, for each observation, a distinct iid sample of size S, $z_i = \left(z_i^{(1)}, \dots, z_i^{(S)}\right)$ for $i = 1, \dots, N$, from the latent model structure of $X|\theta$ in (11). These samples can be used to compute the following unbiased estimator:

$$\widehat{f}_{simple}(\theta|y) = \prod_{i=1}^{N} \sum_{s=1}^{S} P\left(Y_i = y_i|X_i = z_i^{(s)}, \theta\right) \Big/ S \;. \quad \text{(S2)}$$

This estimator is highly inefficient though as it requires to draw $S \times N$ elements, called particles, which can be computationally prohibitive. At the same time though, using the same sample of S particles for all N observations would result in a biased estimator [Lee *et al.*, 2017].

We use a recently developed estimator [Lee *et al.*, 2017] which allows us to use the same sample of S elements to estimate (S1) while preserving unbiasedness, where it is required that $S \geq N$. The algorithm proceeds as follows:

1. Sample $z = (z_1, \dots, z_S)$ from $P(X|\theta)$ in (11), and set $l_0 = 1$ and $\mathcal{S} = \{1, \dots, S\}$.

2. For $i = 1, \dots, N$:

   a) for all $s \in \mathcal{S}$, compute $G_{is} = f_N(y_i - \kappa z_s|\mu_\epsilon, \sigma_\epsilon^2)$;

   b) compute $l_i = l_{i-1} \times \sum_{s\in\mathcal{S}} G_{is}/(S - i + 1)$;

   c) sample w from (S3) and update $\mathcal{S} = \mathcal{S}\backslash\{w\}$.

3. Set $\widehat{f}(\theta|y) = l_N$.

Above, $f_N(.|\mu,\sigma^2)$ denotes the density of the normal distribution with mean μ and variance σ^2. At every iteration of 2), in step 2.c), one particle is sampled, and then removed from $\mathcal{S}$, according to:

$$P(w = s|z, \mathcal{S}) = \begin{cases} \dfrac{G_{is}}{\sum_{s\in\mathcal{S}} G_{is}} & \text{if } i \in \mathcal{S} \text{ and } \sum_{s\in\mathcal{S}} G_{is} > 0, \\ \dfrac{1}{\#\mathcal{S}} & \text{if } i \in \mathcal{S} \text{ and } \sum_{s\in\mathcal{S}} G_{is} = 0, \end{cases} \tag{S3}$$

where $\#\mathcal{S}$ indicates the number of elements in $\mathcal{S}$.

S1.2 MCMC algorithm

We infer the parameters of our model via a Metropolis-within-Gibbs algorithm [Metropolis and Ulam, 1949, Metropolis *et al.*, 1953, Hastings, 1970]: in each iteration of the MCMC, we alternately sample the hierarchical and hyperparameters from their conditional distributions, as illustrated below.

The hyperparameters $\boldsymbol{\Theta}_1, \ldots, \boldsymbol{\Theta}_4$ are sampled from a Gibbs sampler, while $\boldsymbol{\Theta}_5$ is sampled from a Metropolis-Hasting due to the truncated prior for κ. Note that, for μ_5 and τ_5, the proposal and target distributions are very close and so the respective acceptance rates are almost always 1. The measurement error hyperparameters are not sampled since we use a constant informative prior as explained in Section S2.2.

After initialising the parameters in $\boldsymbol{\Theta}$ and $\boldsymbol{\theta}$, we update them according to the following scheme for R iterations.

For $r = 1, \ldots, R$:

Update $\boldsymbol{\Theta}|\boldsymbol{\theta}$: a) For $j = 1, \ldots, 4$ (Gibbs sampler):

$$\mu_j|\tau_j, \boldsymbol{\theta} \sim \mathcal{N}\left(\frac{\tau_j/b_j\, a_j + \tau_j \sum_{k=1}^4 log\left(\theta_j^{(k)}\right)}{\tau_j/b_j + 4\,\tau_j}, (\tau_j/b_j + 4\,\tau_j)^{-1} \right), \tag{S4}$$

with $\mathcal{N}(\mu,\sigma^2)$ being the univariate normal distribution with mean μ and variance σ^2;

$$\tau_j|\mu_j, \boldsymbol{\theta} \sim \mathcal{G}\left(c_j + 4/2, d_j + 1/2 \sum_{k=1}^{4} \left(log\left(\theta_j^{(k)}\right) - \mu_j \right)^2 \right), \tag{S5}$$

with $\mathcal{G}(a,b)$ being the gamma random variable with shape and rate parameters a and b, respectively, i.e. with mean $\dfrac{a}{b}$ and variance $\dfrac{a}{b^2}$.

b) For μ_5 (Metropolis-Hasting sampler), we target the density:

$$\mu_5|\tau_5, \boldsymbol{\theta} \propto p(\mu_5) \prod_{k=1}^{4} p\left(\theta_5^{(k)} \middle| \mu_5, \tau_5\right). \quad \text{(S6)}$$

We propose a move from

$$\mu* \sim \mathcal{N}\left(\frac{\tau_5/b_5\, a_j + \tau_5 \sum_{k=1}^{4} log\left(\theta_5^{(k)}\right)}{\tau_5/b_5 + 4\,\tau_5}, (\tau_5/b_5 + 4\,\tau_5)^{-1}\right), \quad \text{(S7)}$$

and accept it with probability

$$a(\mu*, \mu_5) = \min\left(1, \frac{c(\mu*, \tau_5)}{c(\mu_5, \tau_5)}\right), \quad \text{(S8)}$$

where $c(\mu, \tau) = 1/\int_1^{\infty} f_{log-N}(x|\mu, 1/\tau)dx$, with $f_{log-N}(.|\mu, 1/\tau)$ being the density of the log-normal distribution with mean μ and variance $1/\tau$, and $\min(a, b)$ is the minimum between a and b.

c) For τ_5 (Metropolis-Hasting sampler), we target the density:

$$\tau_5|\mu_5, \boldsymbol{\theta} \propto p(\tau_5) \prod_{k=1}^{4} p\left(\theta_5^{(k)} \middle| \mu_5, \tau_5\right). \quad \text{(S9)}$$

We propose a move from

$$\tau* \sim \mathcal{G}\left(c_5 + 4/2, d_5 + 1/2 \sum_{k=1}^{4} \left(log\left(\theta_5^{(k)}\right) - \mu_5\right)^2\right), \quad \text{(S10)}$$

and accept it with probability

$$a(\tau*, \tau_5) = \min\left(1, \frac{c(\mu_5, \tau*)}{c(\mu_5, \tau_5)}\right). \quad \text{(S11)}$$

Update $\boldsymbol{\theta}|\boldsymbol{\Theta}, \boldsymbol{Y}$: For $k = 1, \ldots, 4$ (Metropolis sampler):

a) We propose a change for the first block of parameters, $\theta_{b1}^{(k)} = \left(log\left(\tilde{\alpha}_0^{(k)}\right), log\left(\tilde{\alpha}_1^{(k)}\right), log\left(\tilde{k}_1^{(k)}\right), log\left(\tilde{k}_0^{(k)}\right), log\left(\kappa^{(k)}\right)\right)$, from the normal distribution:

$$\theta_{b1}^{*} \sim \mathcal{N}(\theta_{b1}, \Sigma_{rb1}^{(k)}), \quad \text{(S12)}$$

where $\Sigma_{rb1}^{(k)}$ is the covariance matrix for the first block of parameters at the r-th iteration of the algorithm. The acceptance probability is defined as:

$$a\left(\theta_{b1}^{*},\theta_{b1}^{(k)}\right)=\min\left(1,\frac{\widehat{f}\left(e^{\theta^{*}}|y^{(k)}\right)\ \pi_{b1}\left(\theta_{b1}^{*}|\mathbf{\Theta}\right)}{\widehat{f}\left(e^{\theta^{(k)}}|y^{(k)}\right)\ \pi_{b1}\left(\theta_{b1}^{(k)}|\mathbf{\Theta}\right)}\right),\qquad \text{(S13)}$$

where $\widehat{f}\left(.|y^{(k)}\right)$ is the unbiased estimate of the marginal likelihood for the k-th replicate, $\pi_{b1}\left(\theta|\mathbf{\Theta}\right)=\prod_{j=1}^{5} f_N(\theta_j|\mu_j,1/\tau_j)$ is the prior for the hierarchical parameters of the first block and $\theta^{*}=(\theta_{b1}^{*},\theta_{b2})$, with θ_{b2} being the parameters of the second block, also in the logarithmic space.

b) We propose a change for the second block of parameters, $\theta_{b2}=\left(log\left(\mu_{\epsilon}^{(k)}\right),log\left(\sigma_{\epsilon}^{(k)}\right)\right)$, from the normal distribution:

$$\theta_{b2}^{*}\sim\mathcal{N}(\theta_{b2},\Sigma_{rb2}^{(k)}),\qquad \text{(S14)}$$

where $\Sigma_{rb2}^{(k)}$ is the covariance matrix for the second block of parameters at the r-th iteration of the algorithm. The acceptance probability is defined as:

$$a\left(\theta_{b2}^{*},\theta_{b2}^{(k)}\right)=\min\left(1,\frac{\widehat{f}\left(e^{\theta^{*}}|y^{(k)}\right)\ \pi_{b2}\left(\theta_{b2}^{*}|\mathbf{\Theta}\right)}{\widehat{f}\left(e^{\theta^{(k)}}|y^{(k)}\right)\ \pi_{b2}\left(\theta_{b2}^{(k)}|\mathbf{\Theta}\right)}\right),\qquad \text{(S15)}$$

where $\pi_{b2}\left(\theta|\mathbf{\Theta}\right)=\prod_{j=6}^{7} f_N(\theta_j|\mu_j,1/\tau_j)$ is the prior for the hierarchical parameters of the second block and $\theta^{*}=(\theta_{b1},\theta_{b2}^{*})$.

In the first 200 iterations, we propose the hierarchical parameters from a simple random walk, where, for $k=1,\ldots,4$, $\Sigma_{rb1}^{(k)}=diag(10^{-6},5)$ and $\Sigma_{rb2}^{(k)}=diag(10^{-6},2)$, with $diag(a,n)$ indicating the $n\times n$ diagonal matrix with a on the main diagonal. We then compute the covariance matrices of the posterior chains in the first and second block which, for the k-th replicate and at the r-th iteration of the MCMC, we define as $Cov_{rb_1}^{(k)}$ and $Cov_{rb_2}^{(k)}$, respectively. After the first 10^5 iterations, which are excluded from the analyses as *burn-in*, we re-compute the covariance matrices excluding the values of the first 0.5×10^5 iterations. The covariances are re-computed every 100 iterations until the end of the MCMC, thus respecting the diminishing

adaptation requirement [Haario *et al.*, 2001]. The covariances are used to compute the respective covariances for the proposal values of the ARW:

$$\Sigma_{rb1}^{(k)} = diag\left(10^{-9}, 5\right) + 0.1 \times Cov_{b_1}^{(k)} \text{ and } \Sigma_{rb2}^{(k)} = diag\left(10^{-9}, 2\right) + Cov_{b_2}^{(k)}, \tag{S16}$$

for $k = 1, \ldots, 4$.

To estimate the marginal likelihood of the data, for $N = 10^3$ data points, we use $S = 10^5$ particles since we noticed, in simulation studies, that this value led to a good mixing of the posterior chains.

As specified in Sections 3 and 4, the algorithm is typically run for $R = 6 \times 10^5$ iterations, of which the first 10^5 are discarded as *burn-in*.

S1.3 Simulation study set-up

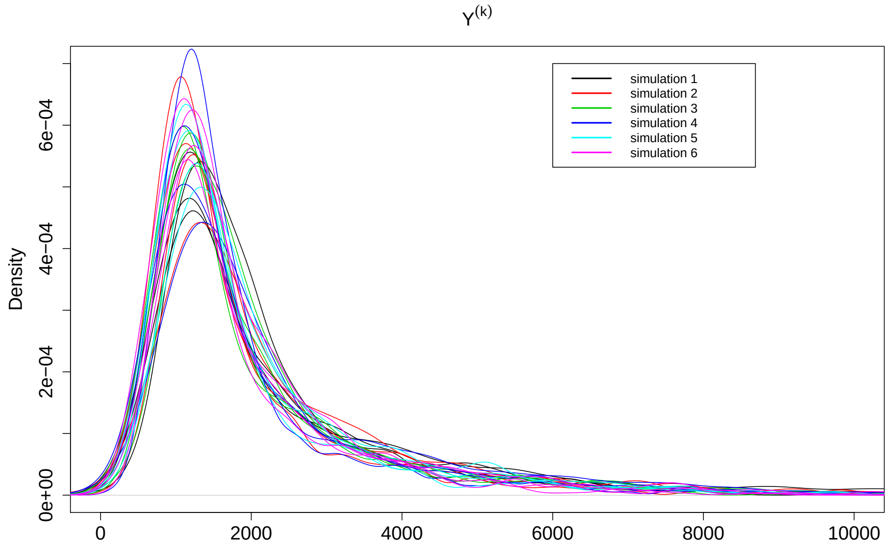

Figure S1: Densities of the simulated data from the six simulation studies.

In this Section, we illustrate the set-up of the simulation study and the parameters used to simulate the data. We carry out six independent simulation studies; in each we sample a dataset whose structure matches the experimental data available: four hierarchical replicates of 1,000 data points each.

In each simulation study, we initially choose a set of hyperprior values, $\mathbf{\Theta}$, and sample 4 independent parameter vectors, $\theta^{(k)} \sim p(.|\mathbf{\Theta})$, $k = 1, ..., 4$. In

particular, each element $\theta_j^{(k)}$ is sampled from a log-normal distribution, i.e. $\theta_j^{(k)} \sim log\mathcal{N}\left(\mu_j, \frac{1}{\tau_j}\right)$, for $k = 1, ..., 4$ and $j = 1, ...5$. The last two elements of $\theta^{(k)}$, i.e. the measurement mean and standard deviation, are kept fixed to their prior mode, i.e. $\mu_\epsilon^{(k)} = e^{\mu_{\mu_\epsilon}^{(k)}}$ and $\sigma_\epsilon^{(k)} = e^{\mu_{\sigma_\epsilon}^{(k)}}$, for $k = 1, ..., 4$, where $\mu_{\mu_\epsilon}^{(k)}$ and $\mu_{\sigma_\epsilon}^{(k)}$ are shown in Table S3.

In the k-th replicate, given $\theta^{(k)} = \left(\tilde{\alpha}_0^{(k)}, \tilde{\alpha}_1^{(k)}, \tilde{k}_1^{(k)}, \tilde{k}_0^{(k)}, \kappa^{(k)}, \mu_\epsilon^{(k)}, \sigma_\epsilon^{(k)}\right)^T$, we independently sample, via equation (11), $P_i^{(k)} \sim Beta\left(\tilde{k}_1^{(k)}, \tilde{k}_0^{(k)}\right)$ and, in turns, $X_i^{(k)} | P_i^{(k)} = p_i^{(k)} \sim Pois\left(\left(\tilde{\alpha}_1^{(k)} - \tilde{\alpha}_0^{(k)}\right) p_i^{(k)} + \tilde{\alpha}_0^{(k)}\right)$, for $i = 1, ..., 10^3$. We then sample the measurement error, $\epsilon_i^{(k)} \sim \mathcal{N}\left(\mu_\epsilon^{(k)}, \sigma_\epsilon^{2\,(k)}\right)$, and obtain the simulated observed value as $Y_i^{(k)} = \kappa^{(k)} X_i^{(k)} + \epsilon_i^{(k)}$, $i = 1, ..., 10^3$. By iterating this scheme on the 4 replicates, we obtain the simulated hierarchical data embedded in $\mathbf{Y} = \left(Y^{(1)}, Y^{(2)}, Y^{(3)}, Y^{(4)}\right)$.

The procedure is repeated independently on each one of the six simulations to obtain six such simulated datasets. The densities for the simulated data for the six simulations, are shown in Figure S1.

The hierchical and hyperparameter values of each simulation study are reported in Tables S1 and S2, respectively; the hyperparameters were chosen to reproduce similar densities to the experimental data.

Simulation	Replicate	$\tilde{\alpha}_0^{(k)}$	$\tilde{\alpha}_1^{(k)}$	$\tilde{k}_1^{(k)}$	$\tilde{k}_0^{(k)}$	$\kappa^{(k)}$	$\mu_\epsilon^{(k)}$	$\sigma_\epsilon^{(k)}$
1	1	20.68	7310.79	0.46	19.13	7.25	775.26	331.27
	2	21.85	8473.88	0.37	21.79	8.31	800.32	309.99
	3	21.44	5912.53	0.43	21.02	8.13	966.62	395.63
	4	18.24	9186.52	0.38	16.42	7.72	892.34	304.54
2	1	19.52	7351.51	0.36	21.51	7.40	775.26	331.27
	2	24.14	7656.17	0.37	18.86	6.86	800.32	309.99
	3	21.60	9247.07	0.40	17.34	7.12	966.62	395.63
	4	20.06	6885.25	0.41	18.51	8.22	892.34	304.54
3	1	21.70	7350.91	0.39	17.38	7.23	775.26	331.27
	2	20.56	7683.30	0.33	19.79	8.19	800.32	309.99
	3	19.97	8199.97	0.35	21.12	7.88	966.62	395.63
	4	20.84	8347.59	0.31	19.38	7.88	892.34	304.54
4	1	16.87	7339.89	0.40	17.05	7.21	775.26	331.27
	2	17.38	9374.83	0.40	22.39	6.78	800.32	309.99
	3	16.52	8457.50	0.37	17.98	7.54	966.62	395.63
	4	18.96	7664.06	0.33	23.86	7.50	892.34	304.54
5	1	21.86	7898.27	0.40	21.75	6.65	775.26	331.27
	2	21.33	8911.15	0.33	19.65	7.34	800.32	309.99
	3	18.25	8179.07	0.39	22.39	7.74	966.62	395.63
	4	15.52	7750.97	0.40	20.53	7.92	892.34	304.54
6	1	19.98	7872.88	0.35	24.01	7.43	775.26	331.27
	2	21.63	8179.49	0.37	19.35	8.78	800.32	309.99
	3	16.44	7618.05	0.41	25.25	6.89	966.62	395.63
	4	22.59	8934.13	0.32	21.14	7.24	892.34	304.54

Table S1: Randomly drawn hierarchical parameter values used in the simulation study.

Parameter	$\tilde{\alpha}_0$	$\tilde{\alpha}_1$	$\tilde{k}_1$	$\tilde{k}_0$	κ	μ_ϵ	σ_ϵ
μ	3	9	-1	3	2	6.75	5.81
τ	10	10	10	10	10	10	10

Table S2: hypermean (first row) and hyperprecision (second row) values used in all six simulation studies to generate the hierarchical parameters shown in Table S1.

S2 Experimental data

S2.1 Measurement process

In this Section we graphically illustrate the measurement process, as described in Section 4.1, via Figure S2.

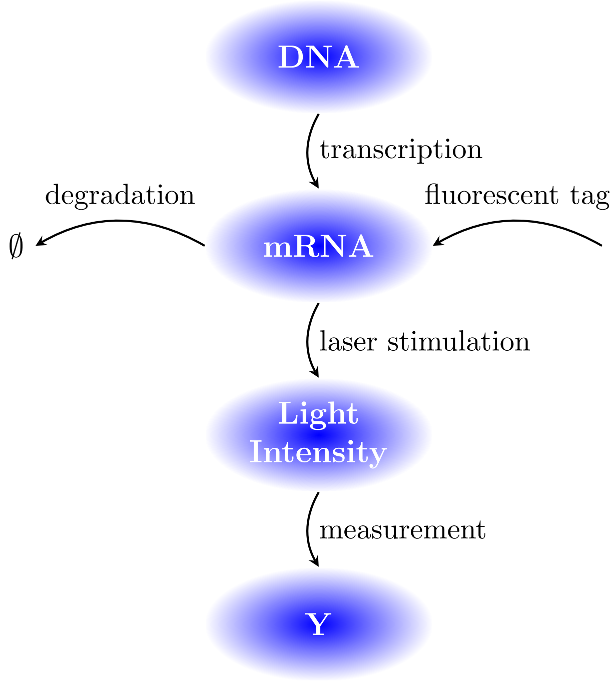

Figure S2: Measurement process for the mRNA population.

S2.2 Exploratory analysis of the measurement error

This Section focuses on a preliminary study of background noise data, which allow us to infer the measurement error parameters and, in turn, to formulate an informative prior for the error mean and standard deviation in each replicate, $\mu_\epsilon^{(k)}$ and $\sigma_\epsilon^{(k)}$, $k = 1, ..., 4$. Since both experimental conditions belonging to the same replicate are measured in the same experiment, it is reasonable to assume, *a priori*, that they share the same measurement error distribution. In other words, the prior obtained on the k-th replicate for the background data is matched with the k-th replicate, for both experimental conditions, for $k = 1, ..., 4$. Indeed, *a posteriori*, we allow all hierarchical parameters to vary in each experimental condition and replicate.

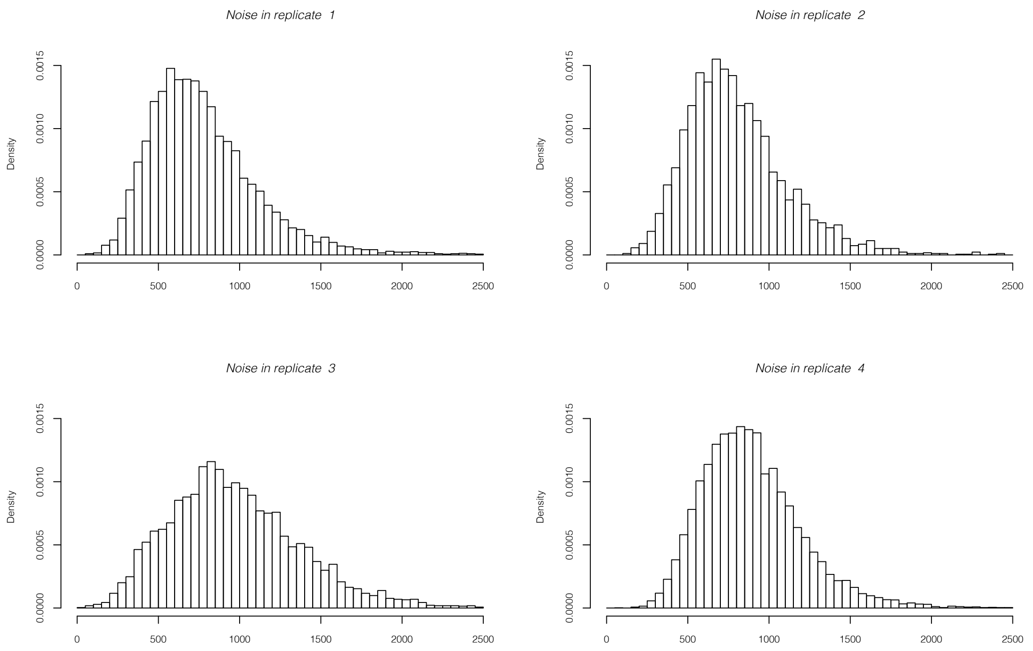

Figure S3: Histograms of the background data in the four replicates.

In order to obtain the background noise data, we use the *CRISPR/Cas9* technology to enzymatically cut out the HIV-1 env gene from the DNA of the observed cells. Then the measurement collection process is performed, identically to the other experimental conditions, as described in Section 4.1. In this way, the population of mRNA is almost absent and the observations approximately correspond to the background noise only; therefore, the measurements are taken as a proxy for the measurement error. The numbers of observations available for the current analysis are 6,257, 3,536, 5,487 and 10,891, for replicates 1 to 4, respectively. It is reasonable to assume that the distribution of the observations for the background of the k-th replicate, which we define as $Z^{(k)} = \left(Z_1^{(k)}, ..., Z_{n_k}^{(k)}\right)^T$, is approximately the same one as for $\epsilon^{(k)}$:

$$Z_i^{(k)} \dot{\sim} \mathcal{N}\left(\mu_\epsilon^{(k)}, \sigma_\epsilon^{2\,(k)}\right), \text{ with } i = 1, \ldots, n_k. \tag{S17}$$

Thus, the posterior densities for the parameters of $Z^{(k)}$, inferred from the background data, are used to formulate informative priors for $\mu_\epsilon^{(k)}$ and $\sigma_\epsilon^{(k)}$. Figure S3 shows the histograms of the background data in the four replicates, which seem to be reasonably well approximated by a normal density.

We implement a Bayesian hierarchical model to infer the measurement error parameters. The conjugate prior distribution choices are the same ones as in the general analysis, as specified in Section S1.2. In particular, *a priori*,

$$\mu_\epsilon^{(k)} \sim log\mathcal{N}\left(\mu_a, \frac{1}{\tau_a}\right) \text{ and } \sigma_\epsilon^{(k)} \sim log\mathcal{N}\left(\mu_b, \frac{1}{\tau_b}\right), k = 1, ..., 4, \tag{S18}$$

where $log\mathcal{N}(a, b)$ denotes the log-normal distribution with mean a and variance b. We assume vague prior distributions for the hyperparameters:

$$\mu_a|\tau_a, \sim \mathcal{N}\left(0, \frac{10^4}{\tau_a}\right), \mu_b|\tau_b, \sim \mathcal{N}\left(0, \frac{10^4}{\tau_b}\right) \text{ and } \tau_a, \tau_b \sim \mathcal{G}(0.001, 0.001). \tag{S19}$$

We define the hyperparameter vector for the analysis of this Section as $\Theta_\epsilon = (\Theta_{\epsilon_a}, \Theta_{\epsilon_b})$, where $\Theta_{\epsilon_a} = (\mu_a, \tau_a)^T$ and $\Theta_{\epsilon_b} = (\mu_b, \tau_b)^T$. We also gather together the hierarchical parameters of all replicates in $\boldsymbol{\mu_\epsilon} = \left(\mu_\epsilon^{(1)}, ..., \mu_\epsilon^{(4)}\right)^T$ and $\boldsymbol{\sigma_\epsilon} = \left(\sigma_\epsilon^{(1)}, ..., \sigma_\epsilon^{(4)}\right)^T$. Figure S4 represents the graphical model associated with the background data.

In order to infer the parameters of this model, we employ a Metropolis-within-Gibbs algorithm [Metropolis and Ulam, 1949, Metropolis *et al.*, 1953, Hastings, 1970], similar to the one described in Section S1.2, where the hierarchical and hyperparameters are alternately sampled from their conditional distributions. Owing to the conjugacy of the hyperprior choices, we use a Gibbs sampler to sample from the posterior distribution of the hyperparameters, conditional on the hierarchical ones:

$$\begin{aligned}
\mu_a|\tau_a, \boldsymbol{\mu_\epsilon}, \boldsymbol{\sigma_\epsilon} &\sim \mathcal{N}\left(\frac{\tau_a \sum_{k=1}^4 log\left(\mu_\epsilon^{(k)}\right)}{\tau_a/10^4 + 4\,\tau_a}, \left(\tau_a/10^4 + 4\,\tau_a\right)^{-1}\right), \\
\tau_a|\mu_a, \boldsymbol{\mu_\epsilon}, \boldsymbol{\sigma_\epsilon} &\sim \mathcal{G}\left(0.001 + 4/2,\ 0.001 + 1/2 \sum_{k=1}^4 \left(log\left(\mu_\epsilon^{(k)}\right) - \mu_a\right)^2\right), \\
\mu_b|\tau_b, \boldsymbol{\mu_\epsilon}, \boldsymbol{\sigma_\epsilon} &\sim \mathcal{N}\left(\frac{\tau_b \sum_{k=1}^4 log\left(\sigma_\epsilon^{(k)}\right)}{\tau_b/10^4 + 4\,\tau_b}, \left(\tau_b/10^4 + 4\,\tau_b\right)^{-1}\right), \\
\tau_b|\mu_b, \boldsymbol{\mu_\epsilon}, \boldsymbol{\sigma_\epsilon} &\sim \mathcal{G}\left(0.001 + 4/2,\ 0.001 + 1/2 \sum_{k=1}^4 \left(log\left(\sigma_\epsilon^{(k)}\right) - \mu_b\right)^2\right).
\end{aligned}$$

Instead, the hierarchical parameters are sampled, conditional on the observations and on the hyperparameters, $\boldsymbol{\mu}, \boldsymbol{\sigma}|\Theta_\epsilon, \boldsymbol{Z}$, from a Metropolis sampler

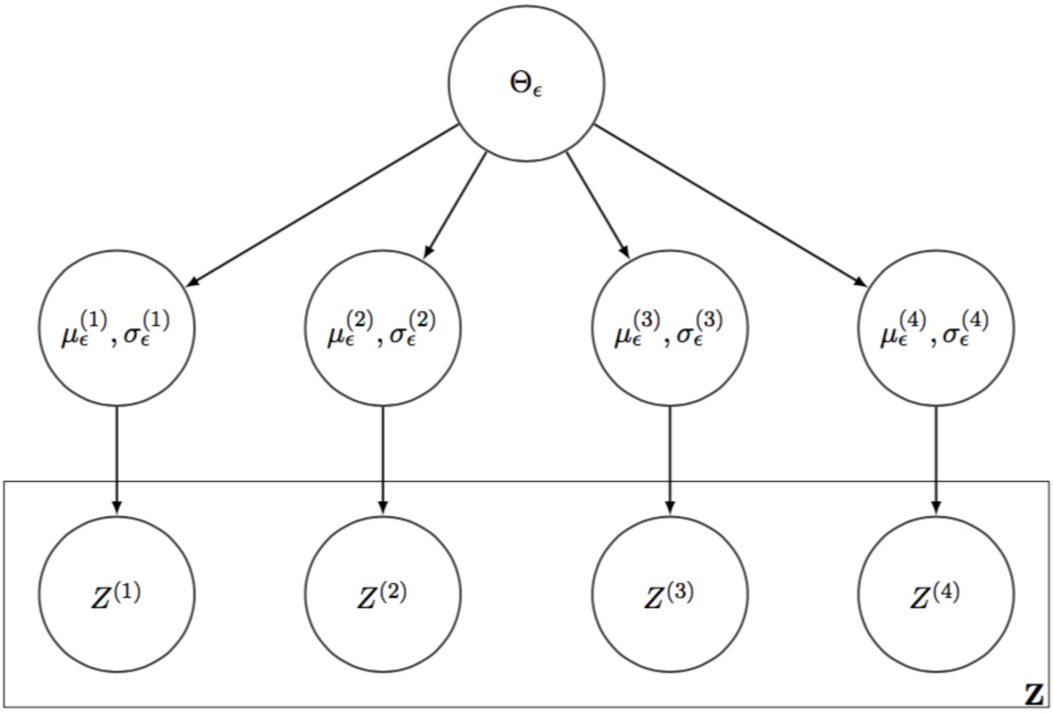

Figure S4: Graphical model for the hierarchical measurement error analysis. On the top of the graph we have the hyperparameters, Θ_ϵ, which generate the hierarchical parameters: from these, the observations $Z^{(1)}, \ldots, Z^{(4)}$ are sampled.

[Metropolis and Ulam, 1949, Metropolis *et al.*, 1953] where the proposals follow an adaptive random walk (ARW) scheme [Haario *et al.*, 2001]. Parameters are sampled, separately for each replicate, in the logarithmic space. In particular, the MCMC is run for $10^5 + 100$ iterations from a random walk with a normal proposal before computing, in each replicate, the covariance of the chains for $log\left(\mu_\epsilon^{(k)}\right)$ and $log\left(\sigma_\epsilon^{(k)}\right)$, excluding the first 10^5 values, which, at the r-th iteration of the MCMC, we call $\Sigma_{ARW}^{(kr)}$. The covariances are then re-computed every 50 iterations, always excluding the initial 10^5 iterations, hence respecting the diminishing adaptation criterion [Haario *et al.*, 2001]. These covariances are used to build the covariance matrix for the proposal values in the Metropolis algorithm, which, for the k-th replicate and at the r-th iteration is $\Sigma_{prop}^{(kr)} = diag\left(10^{-9}, 2\right) + 0.01 \times \Sigma_{ARW}^{(kr)}$, where $diag(a, 2)$ indicates the 2×2 diagonal matrix with a on the main diagonal.

We apply this algorithm to our background data: chains are run for

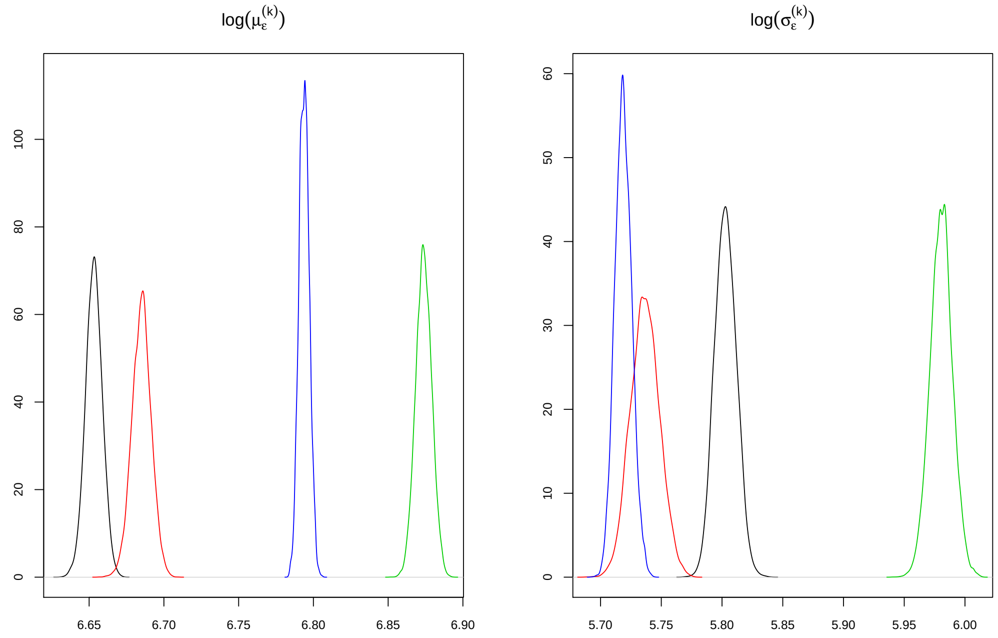

Figure S5: Posterior densities for $log\left(\mu_\epsilon^{(k)}\right)$ (left plot) and $log\left(\sigma_\epsilon^{(k)}\right)$ (right image), $k = 1, ..., 4$. The lines coloured in black, red, green and blue refer to replicates 1 to 4, respectively.

5.5×10^5 iterations, of which the initial 2×10^5 iterations are discarded as *burn-in*. The posterior densities for the hierarchical parameters, in the logarithmic space, $log\left(\mu_\epsilon^{(k)}\right)$ and $log\left(\sigma_\epsilon^{(k)}\right)$, are displayed in Figure S5. From each one of these densities, we compute the posterior mean and standard deviation: values are listed in Table S3, in particular $\mu_{\mu_\epsilon}^{(k)}$ and $\sigma_{\mu_\epsilon}^{(k)}$ indicate the posterior mean and standard deviation for $log\left(\mu_\epsilon^{(k)}\right)$, while $\mu_{\sigma_\epsilon}^{(k)}$ and $\sigma_{\sigma_\epsilon}^{(k)}$ represent the posterior mean and standard deviation for $log\left(\sigma_\epsilon^{(k)}\right)$, for $k = 1, ..., 4$.

These values are used, in the analysis in Section 4, as constant informative priors for the measurement error parameters, with a distinct prior for each replicate.

S2.3 Mean and variance of P, X and Y

In this Section we show how, from the parameters in θ, it is possible to derive the mean and variance of P, X and Y, which are very useful pieces of

Parameter	Replicate 1	2	3	4
$\mu_{\mu_\epsilon}^{(k)}$	6.65	6.69	6.87	6.79
$\mu_{\sigma_\epsilon}^{(k)}$	5.80	5.74	5.98	5.72
$\sigma_{\mu_\epsilon}^{(k)}$	$10^{-3}\times$ 5.54	$10^{-3}\times$ 6.45	$10^{-3}\times$ 5.34	$10^{-3}\times$ 3.37
$\sigma_{\sigma_\epsilon}^{(k)}$	$10^{-3}\times$ 8.80	$10^{-3}\times$ 11.85	$10^{-3}\times$ 9.18	$10^{-3}\times$ 6.91

Table S3: Posterior mean and standard deviation for $log\left(\mu_\epsilon^{(k)}\right)$ and $log\left(\sigma_\epsilon^{(k)}\right)$, in replicates $k = 1, ..., 4$.

information. To keep notation simple, we drop the indicators i and k from parameters and random variables.

Given the beta structure of P, as shown in (5), the mean and variance of P are equal to

$$E(P) = \frac{\tilde{k}_1}{\tilde{k}_1 + \tilde{k}_0}, \tag{S20}$$

$$Var(P) = \frac{\tilde{k}_1\,\tilde{k}_0}{\left(\tilde{k}_1 + \tilde{k}_0\right)^2\left(\tilde{k}_1 + \tilde{k}_0 + 1\right)}. \tag{S21}$$

Considering equations (3)-(6), and owing to the independence of A and B, we can write the mean and variance of X as $E(X) = E(A) + E(B)$ and $Var(X) = Var(A) + Var(B)$. Clearly $E(B) = Var(B) = \tilde{\alpha}_0$ follows from the Poisson distribution of B. While we know that [Johnson *et al.*, 2005]:

$$E(A) = (\tilde{\alpha}_1 - \tilde{\alpha}_0)\,\frac{\tilde{k}_1}{\tilde{k}_1 + \tilde{k}_0}, \tag{S22}$$

$$Var(A) = E(A) + \frac{\tilde{k}_1\,\tilde{k}_0\,(\tilde{\alpha}_1 - \tilde{\alpha}_0)^2}{\left(\tilde{k}_1 + \tilde{k}_0\right)^2\left(\tilde{k}_1 + \tilde{k}_0 + 1\right)}. \tag{S23}$$

From the equations above, we can easily obtain the following formulations for the mean and variance of X:

$$E(X) = \tilde{\alpha}_0 + (\tilde{\alpha}_1 - \tilde{\alpha}_0)\,\frac{\tilde{k}_1}{\tilde{k}_1 + \tilde{k}_0}, \tag{S24}$$

$$Var(X) = E(X) + \frac{\tilde{k}_1\,\tilde{k}_0\,(\tilde{\alpha}_1 - \tilde{\alpha}_0)^2}{\left(\tilde{k}_1 + \tilde{k}_0\right)^2\left(\tilde{k}_1 + \tilde{k}_0 + 1\right)}. \tag{S25}$$

Finally, following the measurement equation in (12) and given the independence between the measurement error, ϵ, and the mRNA population, X, the mean and variance of the observations, Y, can be obtained as:

$$E(Y) = \kappa E(X) + \mu_\epsilon, \quad (S26)$$
$$Var(Y) = \kappa^2 E(X) + \sigma_\epsilon^2. \quad (S27)$$

S2.4 Additional Figures and Tables

Figure S6 shows the densities for the experimental data available, i.e. $Y^{(k)}$, for both experimental conditions in all four replicates.

Table S4 shows the average level of the observations, $\bar{Y}^{(k)} = \sum_{i=1}^{N_k} Y_i^{(k)}/N_k$, in every experimental condition, for each of the four replicates $k = 1, \ldots, 4$, while Table S5 reports the 0.95 level highest posterior density (HPD) credible intervals (CIs) for the exponential of the hypermean parameters, which represents the posterior median of the hierarchical parameters.

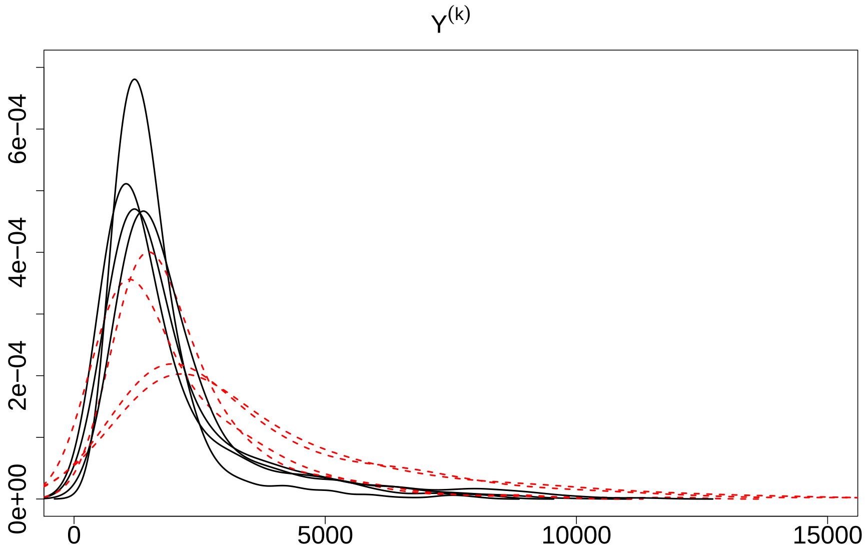

Figure S6: Densities of the experimental data, $Y^{(k)}$. The black solid and red dotted lines refer to cells stimulated with 5 and 10 ng/mL of *tetracycline*, respectively. The four densities per image refer to the four replicates of each experimental condition.

Tables S6-S7 contain the 0.95 level HPD CIs for the hierarchical parameters and informative re-parametrizations. In particular, for every replicate,

	tetracycline	
	5 ng/mL	10 ng/mL
$\bar{Y}^{(1)}$	2000	3778
$\bar{Y}^{(2)}$	1768	1995
$\bar{Y}^{(3)}$	1589	2272
$\bar{Y}^{(4)}$	2273	3518
Average	1907	2891

Table S4: Average level of the observations in the four replicates of each experimental condition.

Parameter	*tetracycline*			
	5 ng/mL		10 ng/mL	
	LB	UB	LB	UB
e^{μ_1}	0.2	184.0	0.0	183.8
e^{μ_2}	145.4	8759.8	64.9	10954.6
e^{μ_3}	0.1	0.4	0.3	0.7
e^{μ_4}	1.9	4.3	2.3	9.6
e^{μ_5}	0.8	22.7	0.9	86.0

Table S5: 0.95 level HPD CIs for the exponential of the hypermean parameters, in both experimental conditions. $e^{\mu_1}, \dots, e^{\mu_5}$ refer to parameters $\tilde{\alpha}_0, \tilde{\alpha}_1, \tilde{k}_1, \tilde{k}_0, \kappa$. "LB" and "UB" represent the limits of the HPD CIs and stand for lower bound and upper bound, respectively.

$k = 1, \dots, 4$, we consider the following elements: the ratio between the transcription in the OFF and in the ON states, $\frac{\alpha_0^{(k)}}{\alpha_1^{(k)}}$, the average time the gene spends in the ON state, $\mu_P^{(k)}$, the average time the gene spends in the OFF state before switching to the ON one, $\frac{1}{\tilde{k}_1^{(k)}}$, the average time the gene spends in the ON state before turning to the OFF one, $\frac{1}{\tilde{k}_0^{(k)}}$, the mean cellular population of mRNA, $\mu_X^{(k)}$, the expected level of the observations, $\mu_Y^{(k)}$, and the ratio between variance and mean of X, $\frac{\sigma_X^{2(k)}}{\mu_X^{(k)}}$.

All HPD CIs are computed in R [R Core Team, 2016] via the *HPDinterval* function of the package *coda* [Plummer *et al.*, 2016].

Figure S7 reports the posterior densities for the four reparametrizations

of the hierarchical parameters: $\mu_X^{(k)}$, $\frac{\sigma_X^{2(k)}}{\mu_X^{(k)}}$, $\mu_Y^{(k)}$, and $\sigma_Y^{(k)}$, with $k = 1, \ldots, 4$. It is clear how the mean and standard deviation of $Y^{(k)}$ observed in the experimental data, denoted by the vertical dotted lines, are always in a central area of the respective posterior density. It is also interesting to note that the ratio between the variance and mean of $X^{(k)}$, i.e. the mRNA latent population of molecules, is very far from 1, which is the assumption of the Poisson model; in particular, the lower bounds of all 0.95 level HPD CIs are bigger than 10.

Figure S8 plots, for each replicate and experimental conditions, the densities obtained in 100 simulations from 100 posterior values of the parameters (represented in green). They closely match the densities of the real data from the corresponding experimental condition (in black), indicating that the parameter values inferred, and the model used, are able to reproduce very similar patterns to the experimental data. Note that the plotted curves are not CIs; nevertheless they provide a visual indication of how well the model can reproduce data compatible with the observed one.

Figure S9 shows the posterior densities for the hyperprecision parameters, $\tau_1, \ldots, \tau_5$, referring to the kinetic parameters $\tilde{\alpha}_0, \tilde{\alpha}_1, \tilde{k}_1$, $\tilde{k}_0$ and κ.

In Figures S10-S11, we plot, for both experimental conditions, the posterior chains, excluding *burn-in*, for the mean and standard deviation of $Y^{(k)}$, i.e. $\mu_Y^{(k)}$ and $\sigma_Y^{(k)}$, $k = 1, \ldots, 4$, and compare them against the empirical values computed on the respective experimental data. All the empirical values, denoted by the horizontal green lines, are in the central area of the respective chain.

Parameter	Replicate 1		Replicate 2		Replicate 3		Replicate 4	
	LB	UB	LB	UB	LB	UB	LB	UB
$\tilde{\alpha}_0^{(k)}$	4.7	205.9	0.9	80.2	4.2	195.2	4.2	209.8
$\tilde{\alpha}_1^{(k)}$	141.1	8591.7	163.6	8471.2	142.9	8390.9	145.6	9470.5
$\tilde{k}_1^{(k)}$	0.2	0.3	0.2	0.3	0.1	0.2	0.2	0.3
$\tilde{k}_0^{(k)}$	1.9	3.8	1.9	3.8	2.1	5.7	2.0	4.2
$\kappa^{(k)}$	1.0	22.9	1.0	20.8	1.0	21.8	1.0	25.6
$\mu_\epsilon^{(k)}$	767.0	783.5	790.2	810.4	957.1	976.7	886.3	898.2
$\sigma_\epsilon^{(k)}$	324.4	335.6	301.9	315.8	386.0	400.2	300.7	308.9
$\frac{\alpha_0^{(k)}}{\alpha_1^{(k)}} \times 10^2$	1.7	3.2	0.4	1.5	1.4	3.3	1.6	2.9
$\mu_P^{(k)} \times 10^2$	7.2	11.5	7.1	11.6	2.8	5.7	7.0	11.4
$\frac{1}{\tilde{k}_1^{(k)}} \times 10^2$	298.3	425.6	298.3	431.0	486.3	913.0	290.9	407.8
$\frac{1}{\tilde{k}_0^{(k)}} \times 10^2$	25.5	50.3	24.6	51.0	15.8	44.1	21.8	45.6
$\mu_X^{(k)}$	21.9	989.2	17.8	840.9	11.7	534.8	17.8	1051.2
$\mu_Y^{(k)}$	1895.7	2082.4	1675.7	1844.3	1544.3	1663.6	2182.3	2394.9
$\frac{\sigma_X^{2(k)}}{\mu_X^{(k)}}$	32.5	1504.9	27.6	1700.9	23.7	1117.7	28.8	1625.3

Table S6: 0.95 level HPD CIs for the hierarchical parameters, and some informative reparametrizations, for cells induced with 5 ng/mL of *tetracycline*.

Parameter	Replicate 1		2		3		4	
	LB	UB	LB	UB	LB	UB	LB	UB
$\tilde{\alpha}_0^{(k)}$	3.4	219.9	0.0	8.4	1.2	99.6	3.1	168.2
$\tilde{\alpha}_1^{(k)}$	103.3	11958.0	66.2	9246.8	83.5	10916.0	80.6	11124.3
$\tilde{k}_1^{(k)}$	0.5	0.7	0.4	0.5	0.3	0.5	0.5	0.6
$\tilde{k}_0^{(k)}$	3.0	8.6	2.6	8.2	3.5	14.6	2.8	7.2
$\kappa^{(k)}$	1.0	99.4	1.0	74.4	1.0	88.5	1.0	85.0
$\mu_\epsilon^{(k)}$	767.2	784.0	789.2	809.1	957.2	977.4	887.0	898.9
$\sigma_\epsilon^{(k)}$	325.7	336.8	300.5	314.3	388.7	402.4	300.2	308.3
$\frac{\alpha_0^{(k)}}{\alpha_1^{(k)}} \times 10^2$	1.0	3.1	0.0	0.3	0.3	1.7	1.0	2.6
$\mu_P^{(k)} \times 10^2$	6.6	14.0	5.1	12.7	2.8	8.6	7.3	14.1
$\frac{1}{\tilde{k}_1^{(k)}} \times 10^2$	140.1	203.6	194.2	259.5	185.4	292.2	154.7	214.6
$\frac{1}{\tilde{k}_0^{(k)}} \times 10^2$	9.9	29.7	10.6	35.2	5.2	23.9	12.3	32.7
$\mu_X^{(k)}$	14.0	1417.4	6.7	810.6	7.4	714.8	14.8	1306.7
$\mu_Y^{(k)}$	3604.2	3956.7	1896.0	2090.7	2185.8	2390.2	3358.1	3677.4
$\frac{\sigma_X^{2(k)}}{\mu_X^{(k)}}$	13.6	1248.9	12.2	1352.9	10.7	998.7	16.3	1326.8

Table S7: 0.95 level HPD CIs for the hierarchical parameters, and some informative reparametrizations, for cells induced with 10 ng/mL of *tetracycline*.

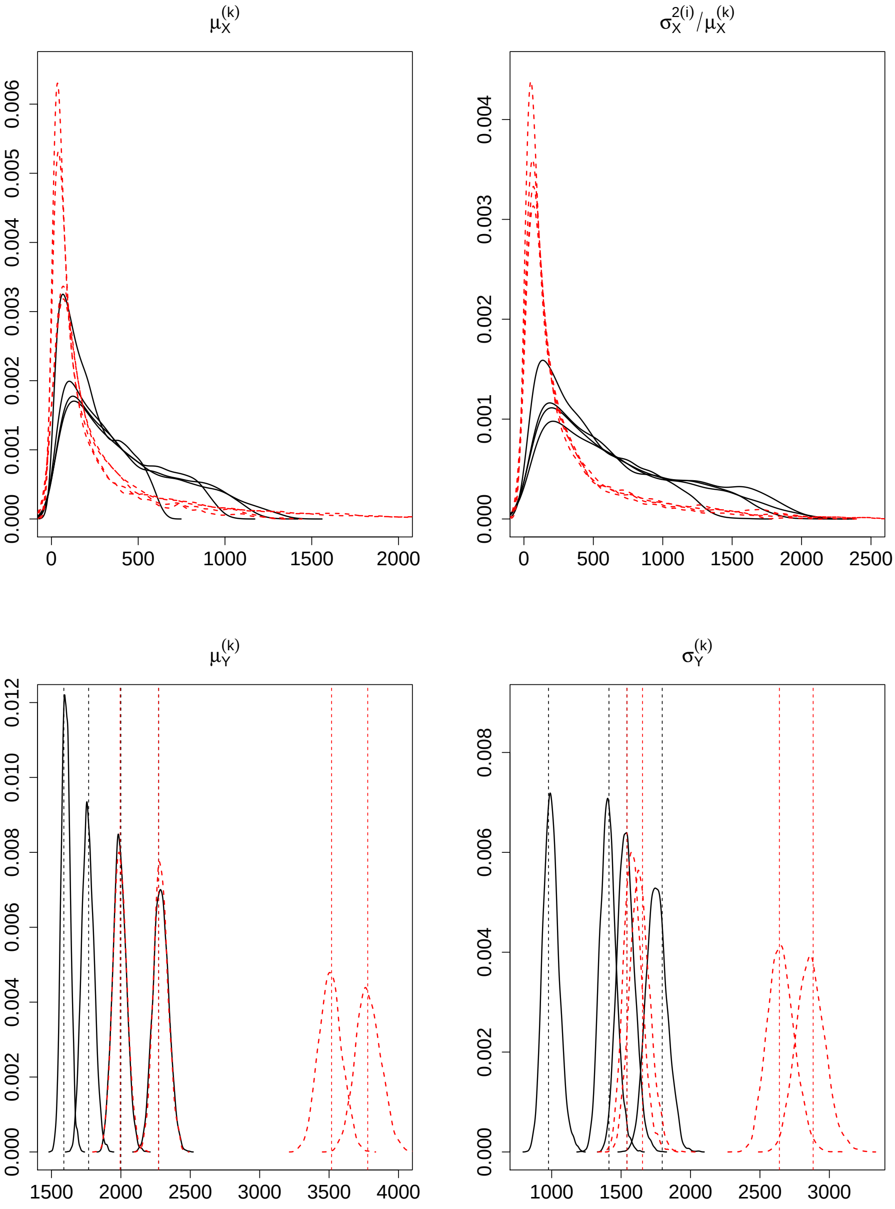

Figure S7: Posterior densities for the following reparametrizations of the hierarchical parameters: $\mu_X^{(k)}$, $\frac{\sigma_X^{2(k)}}{\mu_X^{(k)}}$, $\mu_Y^{(k)}$, and $\sigma_Y^{(k)}$, with $k = 1, \ldots, 4$. The black solid and red dotted lines refer to cells stimulated with 5 and 10 ng/mL of *tetracycline*, respectively. In the bottom images the vertical dotted lines indicate the mean and standard deviation values for $Y^{(k)}$, observed in the experimental data.

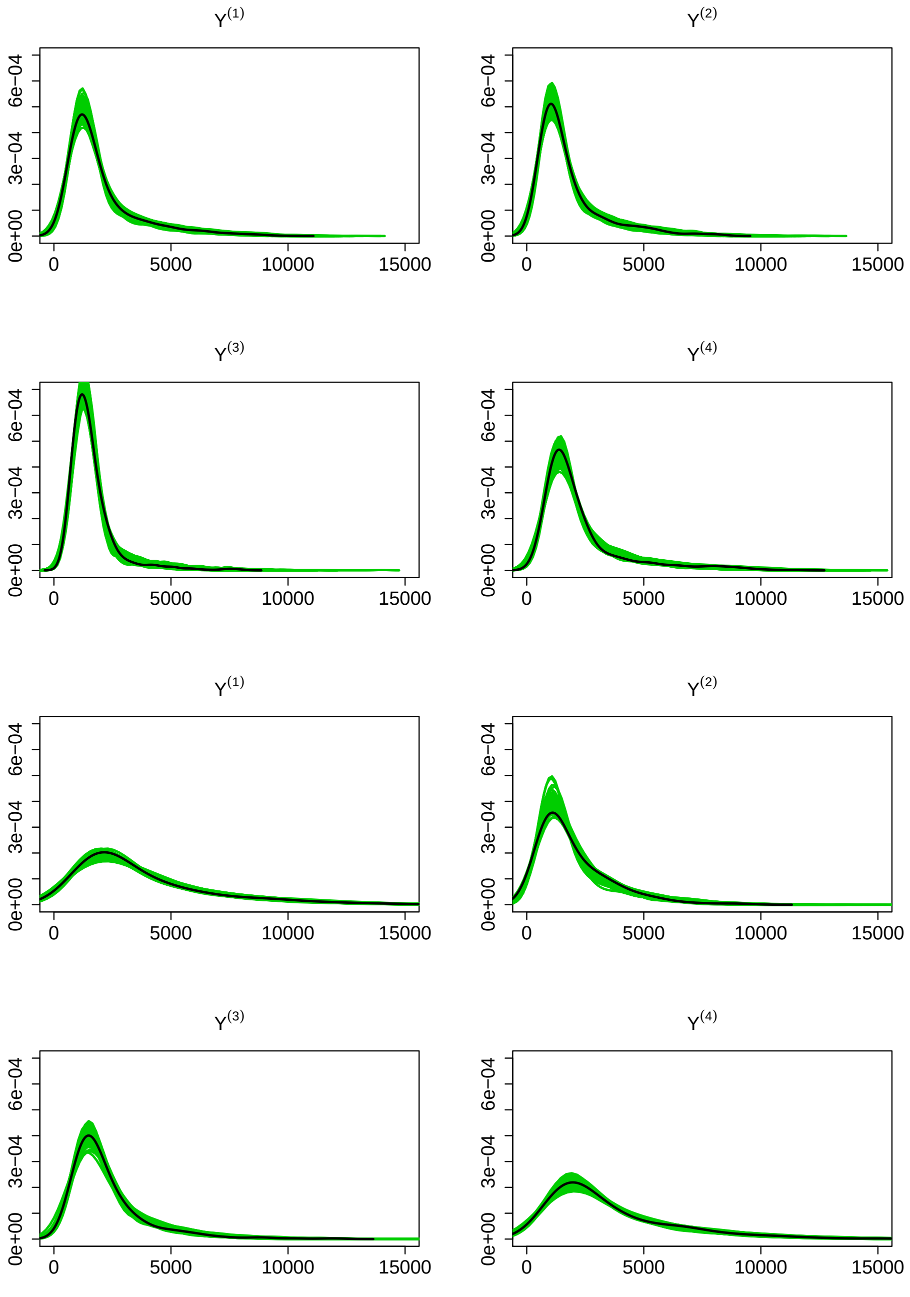

Figure S8: Densities for the data, $Y^{(k)}$, in each replicate $k = 1, \ldots, 4$: in green, the densities for data simulated from 100 posterior values of the parameters; in black, the density for the corresponding experimental data. The bottom and top four plots refer to the cells induced with 5 and 10 ng/mL of *tetracycline*, respectively.

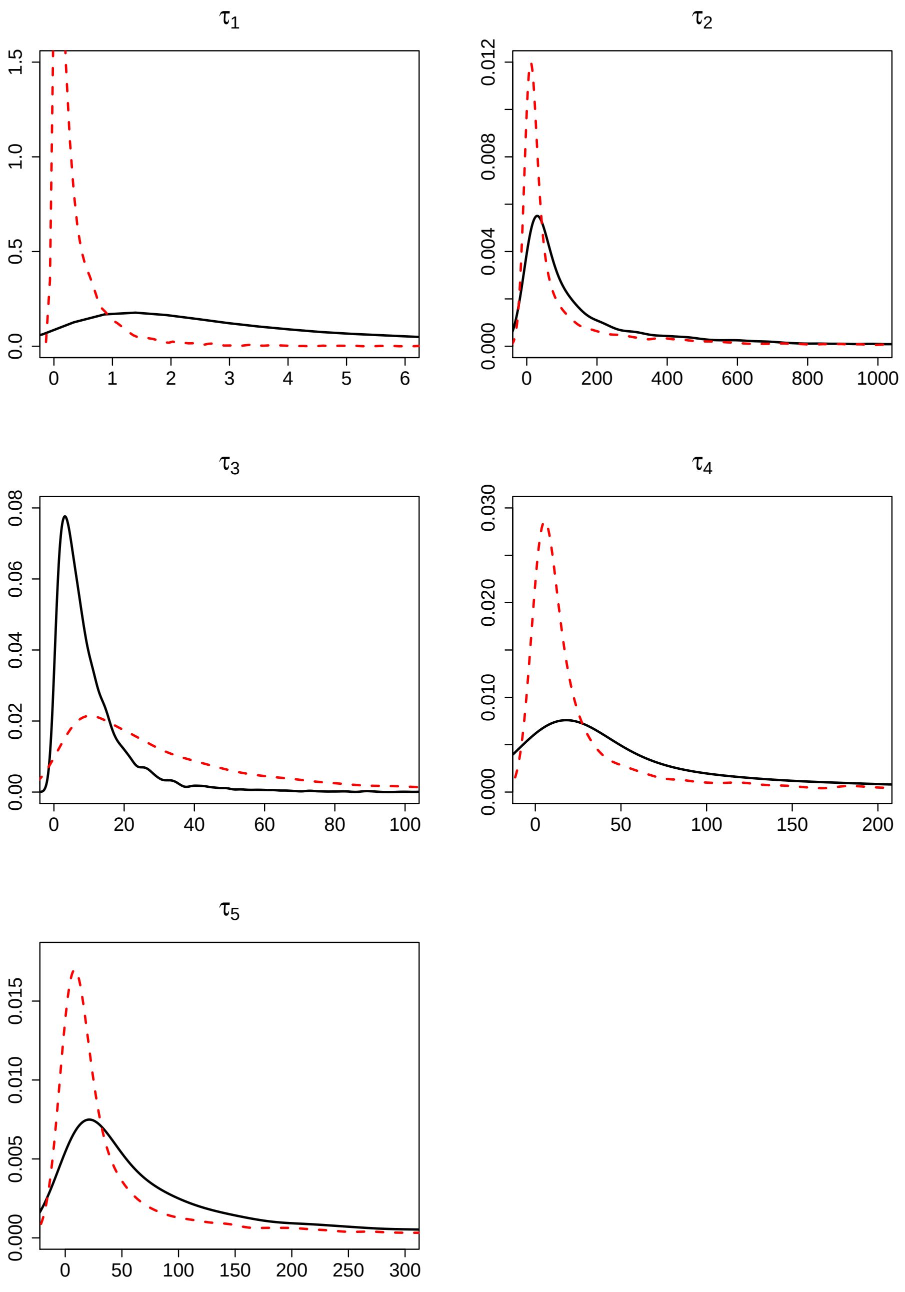

Figure S9: Posterior densities for the hyperprecision parameters, $\tau_1, \ldots, \tau_5$, referring to the kinetic parameters $\tilde{\alpha}_0, \tilde{\alpha}_1, \tilde{k}_1$, $\tilde{k}_0$ and κ. The black solid and red dotted lines refer to cells stimulated with 5 and 10 ng/mL of *tetracycline*, respectively.

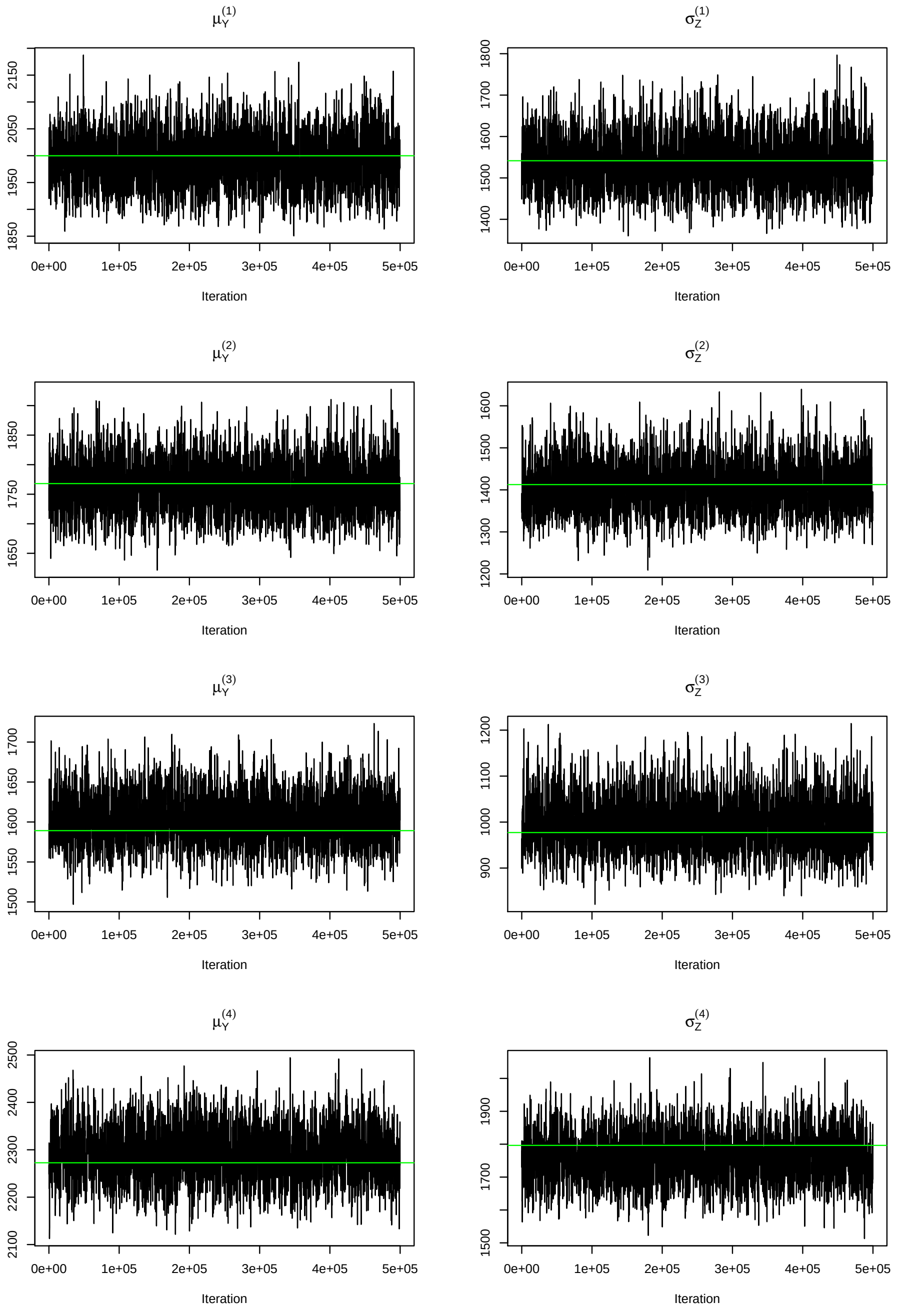

Figure S10: Posterior chains for the mean and standard deviation of $Y^{(k)}$, $k = 1, \ldots, 4$, for cells induced with 5 ng/mL of *tetracycline*. The horizontal green lines represent the empirical mean and standard deviation observed in the respective sample. The chains are thinned: only one value every 100 is plotted.

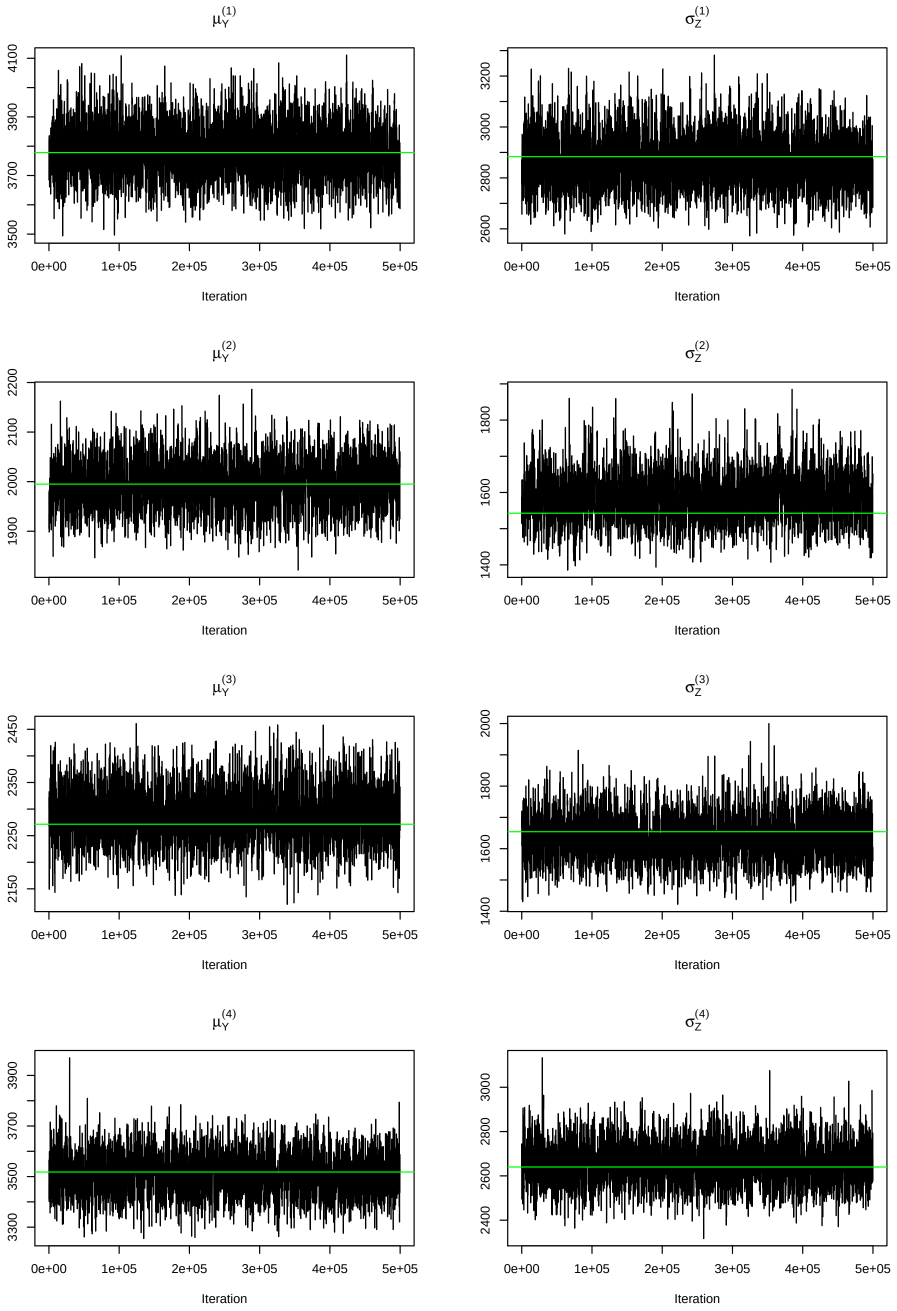

Figure S11: Posterior chains for the mean and standard deviation of $Y^{(k)}$, $k = 1, \ldots, 4$, for cells induced with 10 ng/mL of *tetracycline*. The horizontal green lines represent the empirical mean and standard deviation observed in the respective sample. The chains are thinned: only one value every 100 is plotted.

References

Haario, H. *et al.* (2001). An adaptive Metropolis algorithm. *Bernoulli*, **7**, 223–242.

Hastings, W. K. (1970). Monte carlo sampling methods using Markov chains and their applications. *Biometrika*, **57**, 97–109.

Johnson, N. L. *et al.* (2005). Univariate discrete distributions. *John Wiley & Sons, Hoboken.*

Lee, A. *et al.* (2017). Unbiased approximations of products of expectations. *arXiv:1709.01002*.

Metropolis, N. *et al.* (1953). Equation of state calculations by fast computing machines. *The Journal of Chemical Physics*, **21**, 1087–1092.

Metropolis, N. and Ulam, S. (1949). The Monte Carlo method. *Journal of the American Statistical Association*, **44**, 335–341.

Plummer, M. *et al.* (2016). *coda: output analysis and diagnostics for MCMC.* `https://cran.r-project.org/web/packages/coda/`.

R Core Team (2016). *R: a language and environment for statistical computing*. R Foundation for Statistical Computing, Vienna, Austria. `https://www.R-project.org`.
